# Extensive non-redundancy in a recently duplicated developmental gene family

**DOI:** 10.1186/s12862-020-01735-z

**Published:** 2021-03-01

**Authors:** E. A. Baker, S. P. R. Gilbert, S. M. Shimeld, A. Woollard

**Affiliations:** 1 Department of Biochemistry, University of Oxford, Oxford, OX1 3QU UK; 2 Department of Zoology, University of Oxford, Oxford, OX1 3SZ UK

**Keywords:** Gene duplication, Functional redundancy, Taxon-restricted genes, Hedgehog signalling, Warthog family, *Caenorhabditis elegans*

## Abstract

**Background:**

It has been proposed that recently duplicated genes are more likely to be redundant with one another compared to ancient paralogues. The evolutionary logic underpinning this idea is simple, as the assumption is that recently derived paralogous genes are more similar in sequence compared to members of ancient gene families. We set out to test this idea by using molecular phylogenetics and exploiting the genetic tractability of the model nematode, *Caenorhabditis elegans,* in studying the nematode-specific family of Hedgehog-related genes, the Warthogs. Hedgehog is one of a handful of signal transduction pathways that underpins the development of bilaterian animals. While having lost a *bona fide* Hedgehog gene, most nematodes have evolved an expanded repertoire of Hedgehog-related genes, ten of which reside within the Warthog family.

**Results:**

We have characterised their evolutionary origin and their roles in *C. elegans* and found that these genes have adopted new functions in aspects of post-embryonic development, including left–right asymmetry and cell fate determination, akin to the functions of their vertebrate counterparts. Analysis of various double and triple mutants of the Warthog family reveals that more recently derived paralogues are not redundant with one another, while a pair of divergent Warthogs do display redundancy with respect to their function in cuticle biosynthesis.

**Conclusions:**

We have shown that newer members of taxon-restricted gene families are not always functionally redundant despite their recent inception, whereas much older paralogues can be, which is considered paradoxical according to the current framework in gene evolution.

## Background

Gene duplications are a unique class of mutations in that they act as both substrates and catalysts for evolutionary change. While point mutations, indels and other molecular genetic changes may be acted upon by selection if they affect the fitness of the organism, they cannot do so without altering the pre-existing structure and function of the respective gene. Often, alterations in the sequences of protein-coding genes are deleterious as they impair the already functional protein and the associated phenotype. By comparison, gene and genome duplications provide raw material upon which selection can act, making new evolutionary opportunities possible. Furthermore, in this way, gene duplication can significantly speed up evolution by providing new redundant genetic material that has no constraints and can freely evolve new functions.

Following a gene duplication event, a variety of outcomes are possible. The duplicates may display redundancy with one another, which is considered to be a particularly pervasive genetic phenomenon among recent duplicates [1–3]. Genetic redundancy refers to two or more genes performing the same function, such that the inactivation of one of these genes has no effect on the phenotype [4]. The two Notch-like receptor loci, *lin-12* and *glp-1*, are the products of a recent gene duplication event that occurred during the evolution of the *Caenorhabditis* genus, less than 80 million years ago [5]. Therefore, perhaps unsurprisingly, *lin-12* and *glp-1* are known to be redundant with one another during *C. elegans* embryogenesis [6]. However, functional redundancy may not always be a transient consequence of being recently duplicated but can sometimes persist over longer evolutionary time scales as found in studies of ancient paralogues in budding yeast and nematode worms [7, 8].

Generally, it is thought that complete redundancy between duplicates is unstable in the long term. Rather, duplicated genes are thought to adopt one of three common fates. Firstly, neofunctionalisation is a scenario in which one of the copies acquires a new function relative to the ancestral gene [9]. While neofunctionalisation is thought of as the primary mechanism by which morphological novelty arises, it is considered to be a rare fate of duplicate genes. The second and most common fate of duplicate genes is pseudogenisation, where the relaxed selection on one of the duplicates allows the gene to accumulate null mutations. Thirdly, the duplication-degeneration-complementation (DDC) model supposes that in the event of gene duplication, the two copies degenerate to perform complementary functions that jointly match that of the ancestral gene, a process known as subfunctionalisation [10, 11].

Specialisation is a nuanced take on the classical fates of duplicated genes. It is a form of asymmetric paralogue divergence where one duplicate becomes highly specialised in a distinct aspect of the ancestral gene’s function, while the other retains a broader association with the ancestral function [12, 13]. This newly characterised behaviour of duplicate genes has been poorly assessed in studies of expanded gene families and is rarely investigated using robust molecular genetic techniques.

The Warthogs are a family of Hedgehog-related (Hh-r) genes exclusively found in the nematode phylum and are products of many gene duplication events [14]. Unlike their nematode-specific counterpart, the Hedgehog family has diversified little throughout the Bilateria, with most species possessing only one true orthologue. Two rounds of whole genome duplication have given rise to three genes in vertebrates [Sonic Hedgehog (Shh), Indian Hedgehog (Ihh), Desert Hedgehog (Dhh)], and due to an additional round of whole genome duplication, four or five in ray-finned fish (reviewed by [15]). These vertebrate ohnologues arose approximately 530 million years ago and have taken on distinct, non-redundant, developmental roles. However, one of the teleost-specific ohnologues, *tiggywinkle hedgehog*, is around 350 million years old and appears to be redundant with *shh* in zebrafish retinal development [16]. This study, however, was limited to gene expression pattern analysis so it remains to be established what the knockout phenotypes would be.

Aside from its conservation in some basal nematode species including *Trichuris trichiura*, *Soboliphyme baturini* and *Trichinella zimbabwensis* (see Additional file 3: Table 1), most nematodes have lost a Hedgehog gene. They have instead evolved an expanded repertoire of 61 Hh-r genes with partial orthology to the ‘Hog’ domain, or carboxyl terminus, of Hedgehog proteins. There are no homologues of the ‘Hedge’ domain, or amino terminus of Hedgehog, in the Hh-r superfamily of genes. The absence of the Hedge domain was surprising upon the initial discovery of Hh-r genes, as fly and mammalian Hedgehog pro-peptides are known to be autocleaved in the endoplasmic reticulum by their enzymatic Hog domain, prior to the release of the Hedge domain for signalling and the Hog domain for proteasomal degradation [17, 18]. In other words, the Warthog family only possess partial orthology to the cleaved and degraded portion of the canonical Hedgehog protein. Nevertheless, the novel amino-terminal domains associated with Hog in nematodes were classified initially as Warthog (WRT) and Groundhog (GRD) [14], followed by Ground-like (GRL) and Quahog (QUA) [19]. While all ten Warthogs contain a ‘Wart’ domain (defined by a consensus sequence of eight cysteine residues), only five family members contain a Hog domain: WRT-1, WRT-4, WRT-6, WRT-7 and WRT-8.

To test the relationship between the age of gene duplicates and the likelihood of functional redundancy in the Warthog family, we set out to investigate their roles in the model nematode *C. elegans* by first characterising their evolutionary history in Nematoda. To systematically elucidate their duplication history, we used a combination of molecular phylogenetic algorithms and then knockout and knockdown approaches in *C. elegans* to assess the functional divergence of paralogous genes.

We find the Warthog family clades have neofunctionalised with respect to a handful of post-embryonic developmental processes, including left–right (LR) asymmetry, vulval fate determination and body size regulation. Contrary to expectation, we find no examples of complete redundancy between the more recently derived Warthogs in these neofunctionalised clades, despite many of these genes being restricted to the *Caenorhabditis* genus. We find that seven out of ten family members are involved in aspects of ecdysis, but while five of those exhibit additive moulting phenotypes, two divergent Warthogs belonging to different clades display redundancy with respect to their role in cuticle biosynthesis. Reconciliation of these phenotypes with the phylogeny of the Warthog family suggests that the ancestor of the family was probably pleiotropic—involved in both moulting and cuticle biosynthesis—and though most Warthogs have retained a broad association with the ancestor with respect to moulting (*wrt-1, wrt-2, wrt-4, wrt-5* and *wrt-8*), two genes (*wrt-3* and *wrt-9*) have independently specialised solely in cuticle formation. Overall therefore, we have shown that taxon-restricted gene families are capable of assuming important developmental roles, and that despite being recently derived, most members of the Warthog family are non-redundant with one another in various post-embryonic contexts.

## Results

### Widespread gene duplications in the Warthog family

We mined the predicted proteomes of a phylogenetically diverse range of nematodes for the Wart domain and verified the hits individually to ensure they contained a *bona fide* Wart domain as defined by Bürglin [14]. We exclusively analysed the Warthog repertoires of major parasites and model organisms so as to prevent conclusions about gene family evolution being an artefact of genome quality or the completeness of predicted proteomes [20]. Nematoda is divided into three lineages, namely, Enoplea, Dorylaimia, and Chromadorea, although orders are commonly organised into five major clades that do not correspond to the divisions of classical taxonomy [21]. The following species were selected for molecular phylogenetic analyses: *Brugia malayi* (Clade III),*Toxocara canis* (Clade III),*Ascaris suum* (Clade III),*Strongyloides ratti* (Clade IV); *Pristionchus pacificus* (Clade V); *Caenorhabditis remanei* (Clade V); *Caenorhabditis brenneri* (Clade V); *Caenorhabditis briggsae* (Clade V) and *Caenorhabditis elegans* (Clade V). Multiple species from Clade I were selected as outgroups (*Trichinella spiralis*, *Trichinella nativa*, *Trichinella murelli*, *Trichinella sp. T6*, *Trichinella sp. T8*, *Trichinella sp. T9*, *Trichinella papuae*, *Trichinella patagoniensis*, *Trichinella nelsoni*, *Trichinella pseudospiralis*, and *Trichuris suis*) as these were the only species in which only a single Wart domain could be detected. *Trichinella zimbabwensis*, *Trichuris trichiura* and *Soboliphyme baturini* were all found to contain at least one Hedgehog homologue, yet no Warthog homologues could be detected. We attempted to use *Trichuris muris* and *Romanomermis culcivorax* as other Clade I/Enoplea representatives in our analyses but no Hedge/Wart/Ground/Qua/Hog/Ground-like domain sequences could be detected in their predicted proteomes. As it was considered very unlikely for a bilaterian animal to have lost all Hedgehog and/or Hedgehog-like genes given their presence in neighbouring lineages, the genomes of *T. muris* and *R. culcivorax* were not deemed to be of sufficient completeness for use in our investigation.

**Fig. 1:**
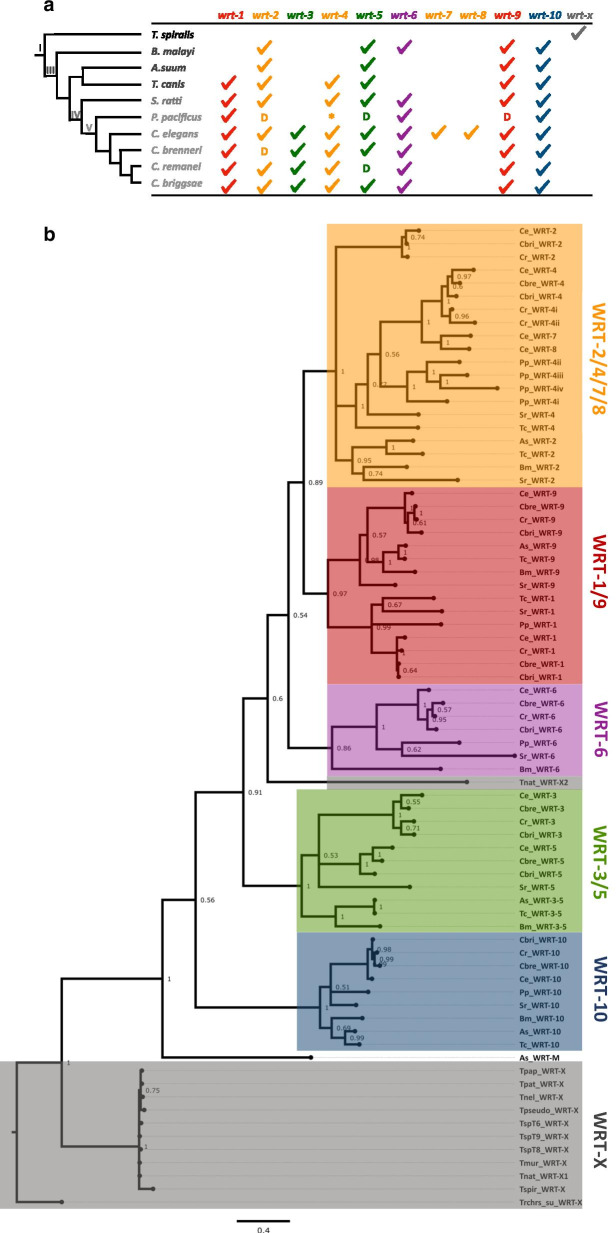
Phylogenetic analysis of the Warthog family in *C. elegans* and other nematodes. **a** Cladogram showing relationships between nematodes in this study and a table showing their Warthog orthologues. Coloured ticks indicate that Warthog is present in a respective species. ‘D’ refers to degenerate Wart domain sequences. ‘*’ refers to the abnormal *wrt-4* complement in *Pristionchus pacificus* which has four *bona fide wrt-4* orthologues and four degenerate *wrt-4* sequences. **b** Phylogram was generated from a multiple sequence alignment of Wart domains (Additional file 1: Fig. 1), including *C. elegans* paralogues (stars) and orthologues from selected nematode species. Wart clades are colour coded. Species abbreviations: *Tnat, Trichinella nativa*; Tmur, *Trichinella murelli*; TspT6, *Trichinella sp. T6*; TspT8, *Trichinella sp. T8*; TspT9, *Trichinella sp. T9*; Tpap, *Trichinella papuae*; Tpat, *Trichinella patagoniensis*; Tnel, *Trichinella nelsoni*; Tpseudo, *Trichinella pseudospiralis*; Trchrs_su, *Trichuris suis;* Ts, *Trichinella spiralis*; Bm, *Brugia malayi*; As, *Ascaris suum*; Tc, *Toxocara canis*; Sr, *Strongyloides ratti*; Pp, *Pristionchus pacificus*; Cbre, *Caenorhabditis brenneri*; Cbri, *C. briggsae*; Cr, *C. remanei*; Ce, *C. elegans*. ‘As_WRT-M’ is our given name to the Warthog in *A. suum* which did not robustly cluster into any of the Wart clades. Node values indicate posterior probabilities for each split. The scale bar indicates average branch length measured in expected substitutions per site

Figure 1a summarises the Warthogs present in the nematodes analysed. Mining the genomes of these nematodes for Wart domains revealed multiple hits which had partially lost the consensus sequence (one or more cysteine residues) but otherwise aligned to one of the ten *C. elegans* Wrts. Because they had incompletely lost a typical Wart domain sequence, we classified them as ‘degenerate Wrts’. In most cases, degenerate Wrt coding sequences have diverged by more than just their cysteine residues which probably reflects their neofunctionalisation outside of Warthog niches, except for the *wrt-2* orthologues in *C. brenneri* and *C. remanei* which have accumulated a large proportion of repetitive and low complexity DNA.

Two independent phylogenetic analyses were run on the Wart domain alignment (see Additional file 1: Fig. 1). The output of the Bayesian analysis is shown in Fig. 1b (the maximum likelihood IQ-TREE analysis can be found in Additional file 2: Fig. 2). Wart domain sequences from other nematodes were named because of their similarity to *C. elegans* sequences (such that the ten *C. elegans* Warthogs remained the basis of this investigation). Since there are more loci in other nematodes than previously named, we propose an updated Warthog nomenclature based on the Wart domain Additional file 3: Table 1).

Both phylogenetic analyses resolved five distinct Wrt clades: WRT-2/4/7/8 (containing WRT-2, WRT-4, WRT-7, WRT-8); WRT-3/5 (containing WRT-3, WRT-5); WRT-1/9 (containing WRT-1, WRT-9); WRT-6 (containing WRT-6 only); WRT-10 (containing WRT-10 only). The single Wart domains in Clade I nematodes root both phylogenetic trees and are taken as the extant representative of the ancestral Wart domain. ‘As_WRT-M’ is our name given to the Warthog in *A. suum* which did not robustly cluster into any of the Wrt clades.

Unusually, *P. pacificus* contained four *bona fide* ‘WRT-4’ orthologues (Pp_WRT-4i, Pp_WRT-4ii, Pp_WRT-4iii, Pp_WRT-4iv) and three degenerate ‘WRT-4’ sequences. Only Pp_WRT-4iii possesses a Hog/Hint domain, while all other paralogues do not Additional file  4: Fig. 3), which may suggest only part of the locus is prone to duplicate. An alternative explanation may be inaccurate protein prediction models [20]. The atypical *wrt-4* complement in *P. pacificus* was found to be species-specific but is probably symptomatic of the gene’s repetitive content. The genome instability conferred by repetitive sequences [22] and their tendency to cause the duplication of adjacent regions means that tandem and inverted repeats provide opportunities for gene duplication by providing regions of homology for unequal crossing over. Throughout this investigation, we noticed an abundance of tandem and inverted repeats in and around *C. elegans* Wrt gene sequences, later mined using RepeatMasker (unpublished observations). As it is known that repetitive elements are similarly distributed on *C. elegans* autosomes [23], and as all Warthog genes contain introns, we propose that all family members have been derived by unequal crossing over as opposed to retrotransposition.

To further probe into the duplication history of these genes, we performed synteny analysis Additional file 5: Table 2). The extent of genomic reshuffling even within the *Caenorhabditis* genus meant this strategy was not as useful for characterising gene family evolution compared to its illumination of gene diversification in chordates [24] wherein synteny is more highly conserved. In all clade V nematodes *wrt-1* and *wrt-10* were 350 bp apart yet in *S. ratti* and *T. canis* they were on different chromosomes, most likely because of lineage-specific reshuffling. The two *C. elegans* specific Warthogs, *wrt-7* and *wrt-8*, were directly adjacent to one another on chromosome V and their loci map to *wrt-4* in other Rhabditina. Outside of clade V (e.g., *S. ratti*, *A. suum*, *T. canis*, *B. malayi*, *T. spiralis*), many microsyntenic relationships break down.

### Functions of the *C. elegans* Wrt genes strongly associate with clades of the Warthog phylogeny

#### Members of the Wrt-2/4/7/8 clade are involved in the development of LR asymmetry

In order to investigate the possible redundancy relationships among duplicated Wrt genes, we first tested the phenotypes of single knockdowns (by RNAi) and single knockouts (using deletion alleles), and later double and triple mutants. All phenotypes reported in this study are confirmed by both knockout and knockdown approaches to increase reliability.

**Fig. 2:**
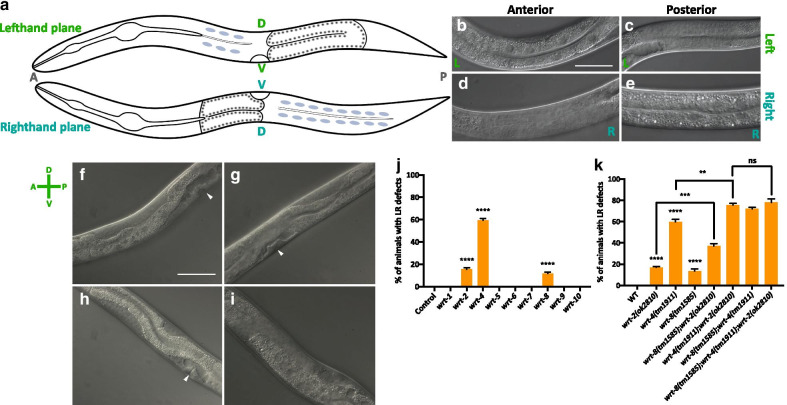
Presence of middle body LR asymmetry defects in Wrt family knockdowns and knockouts. **a** Schematic of a wild-type worm in both dorsal and ventral views showing the gut/gonad asymmetry. **b–e** Wild-type images of the middle body where **b** and **c** are taken in the lefthand plane and **d** and **e** are taken in the righthand plane. Intestine (**b** and **e**) is recognisable for the large nuclei and the gonad (**c** and **d**) is most recognisable for being syncytial. **f**–**i** *wrt-2(ok2810)* animals exhibiting defects in middle body LR asymmetry, where in **f** and **h** (lefthand plane) only intestine should be visible yet patches of gonad are observed. In **g** and **i** (righthand plane), only gonad should be visible, yet patches of gut are observed. Arrowheads indicate vulvas. Scale bars 50 μm. **j** % penetrance of LR defects upon knocking down a Wrt family member (x-axis). Empty vector control (L4440) animals displayed no defects in the positioning of their gut and gonads relative to one another (n = 51), nor did *wrt-1* (n = 80); *wrt-5* (n = 71); *wrt-6* (n = 45); *wrt-7* (n = 67); *wrt-9* (n = 85) or *wrt-10* (n = 59) RNAi animals. *wrt-2* (n = 72), *wrt-4* (n = 80) and *wrt-8* (n = 56) RNAi animals did display LR asymmetric defects. *wrt-3* knockdown results in animals with miniaturised or absent gonad arms and/or other disruptions to their middle body anatomy such that LR defects could not be quantified in *wrt-3* defective animals (Additional file 8: Fig. 5). (K) % penetrance of LR defects in wild-type as compared to animals carrying the *wrt-2(ok2810)* allele (n = 65), the *wrt-4(tm1911)* allele (n = 108), the *wrt-8(tm1585)* allele (n = 68) or the following double/triple mutants: *wrt-4(tm1911)*;*wrt-2(ok2810)* (n = 102); *wrt-8(tm1585)*;*wrt-2(ok2810)* (n = 87); *wrt-8(tm1585)*;*wrt-4(tm1911)* (n = 54); *wrt-8(tm1585)*;*wrt-4(tm1911)*;*wrt-2(ok2810)* (n = 103)*.* Black bars show mean + SEM (J, K). Black asterisks (****P ≤ 0.0001, ***P ≤ 0.001, **P ≤ 0.01, *P ≤ 0.05, nsP > 0.05) show statistically significant differences in the means compared to Control RNAi with an unpaired *t* test (J) or in the means of Wrt mutants compared to WT with an unpaired *t* test (**k**)

Upon initial investigation, it was noted that the characteristic orientation of the gut and gonad with respect to one another was disrupted in *wrt-2(ok2810)* mutant animals. In wildtype (WT) worms, there is an invariant left–right (LR) asymmetry in the middle body Fig. 2a where in the lefthand plane only intestine is visible in the anterior Fig. 2b and only gonad arm is visible in the posterior Fig. 2c. Conversely in the righthand plane, only gonad is seen anteriorly Fig. 2d, while only intestine is seen posteriorly Fig. 2e. Examples of deviations from the WT presentation in *wrt-2(ok2810)* animals are shown in the lefthand plane in both the anterior Fig. 2f, h and the posterior Fig. 2g, i,only gut or gonad should be observed respectively, yet both are seen (to variable extents) in the same plane. No other obvious gonad morphology defects were observed in these animals, for example aberrant turns or projectiles (lateral guidance defects) normally associated with dorsoventral (DV) or anteroposterior (AP) axis misguidance. Thus *wrt-2* appears to be involved in specifically regulating LR asymmetry in the middle body of the adult worm.

In order to test whether knockdown of other Wrt genes produces a similar phenotype we performed RNAi knockdown of each family member and recorded the penetrance of defects in the middle body of the worm compared to empty vector control RNAi animals Fig. 2j. We found that only knocking down *wrt-2*, *wrt-4* or *wrt-8* resulted in LR asymmetric defects with *wrt-4* knockdown resulting in the highest penetrance of 60% (P < 0.0001). Knockdown of *wrt-2* and *wrt-8* gives rise to 16% (P < 0.0001) and 12% (P < 0.0001) of animals with LR defects, respectively. Thus, all members of the Wrt-2/4/7/8 clade display LR defects upon RNAi knockdown except *wrt-7*. To confirm this, we analysed the phenotypes of *wrt-2(ok2810)*, *wrt-4(tm1911)* and *wrt-8(ok1585)* single mutants, finding concordance with the RNAi data Fig. 2k. Next, we tested phenotypic redundancy between Wrt-2 clade members by constructing double and triple mutants and quantifying the penetrance of LR asymmetric defects. We observed the penetrance of defects in the *wrt-2*,*wrt-8* (P = 0.0003), *wrt-2*,*wrt-4* (P = 0.0025), *wrt-4*;*wrt-8* double mutants to be additive suggesting these pairs of genes do not display redundancy with respect to this phenotype. Moreover, the concomitant inactivation of *wrt-2*, *wrt-4* and *wrt-8* in the triple mutant did not increase the penetrance of LR defects over and above the *wrt-4;wrt-2* double mutant (P = 0.5478).

It is worth noting that performing *wrt-7* RNAi-mediated knockdown on *wrt-2(ok2810), wrt-4(tm1911)* and *wrt-8(ok1585)* single mutants and the inverse set of experiments (i.e. *wrt-2, -4* and *-8* RNAi knockdown on *wrt-7(ok3271)* mutant animals) did not reveal a role for *wrt-7* in any obvious biological process. This includes the absence of defects in LR asymmetry in the middle body as there were no phenotypic differences between these and the relevant control animals Additional file 6: Fig. 4). In addition, *wrt-7* RNAi knockdown in an RNAi-sensitive mutant (*rrf-3(pk1426)*) did not display any abnormal morphologies when compared to control animals Additional file 6: Fig. 4). Taken together with reports that *wrt-7* is not expressed throughout development (from PolyA + and Ribozero modENCODE libraries [25, 26]), we conclude that *wrt-7* is non-functional and has likely pseudogenised. Although the hallmarks of pseudogenisation (e.g. a premature stop codon) are absent in the Bristol N2 strain, many wild isolates of *C. elegans* contain a highly polymorphic copy of *wrt-7* that includes a missing start codon and approximately 50 moderate effect mutations Additional file 7: Table 3).

Despite the clear roles of *wrt-2*, *wrt-4* and *wrt-8* in the establishment of LR asymmetry during late larval development in the middle body, we were not able to detect embryonic defects (in either early embryos at the four-to-six cell stage, when LR asymmetry is established in *C. elegans* embryos, or during the intestinal twist at the 1.5-fold stage of mid-embryogenesis) in left–right asymmetry which would have suggested that these genes are global regulators of LR asymmetry (data not shown). Therefore, we infer that these genes are unique in providing a left–right directional signal for the gonad arms as they migrate along the AP and DV axes during larval development (reviewed by [27]). No signals were previously implicated in the left–right guidance of gonad morphogenesis because it was considered to be a consequence of AP and DV signalling by molecules such as netrin [28]. Notably, *wrt-4*, *wrt-2* and *wrt-8* must not be the only regulators of this aspect of left–right positioning, as no animals were seen with complete reversals of middle body morphology, known as situs inversus, implying other signals are required for this process. Nevertheless, it is striking that members of the Warthog family are involved in the generation of LR asymmetry given the well characterised role of the partially orthologous Shh in the same process during mammalian embryogenesis [29].

#### Members of the Wrt-3/5 clade are involved in cell fate determination in the developing vulva

**Fig. 3:**
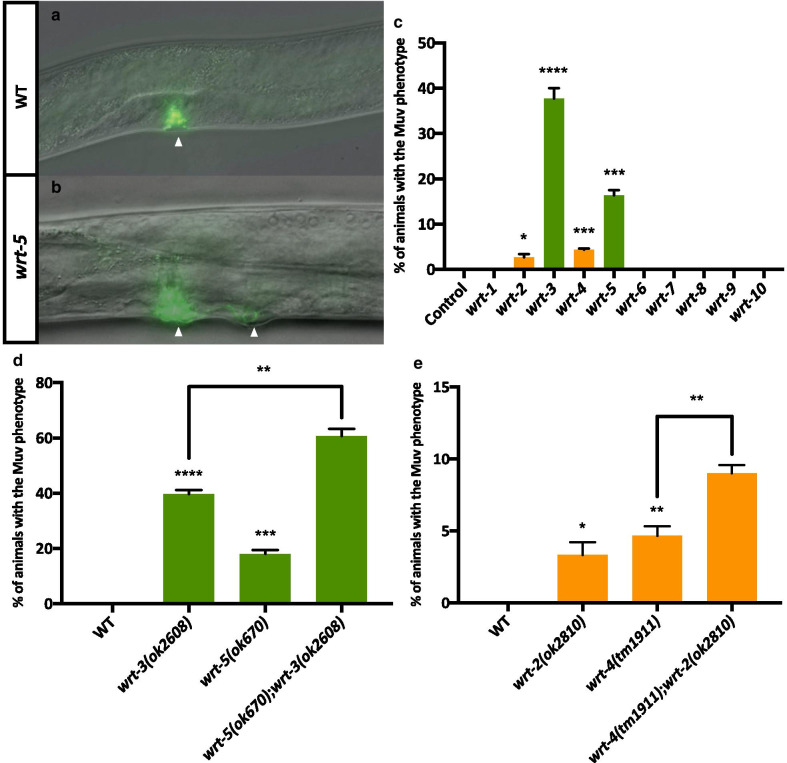
Presence of the Multivulva phenotype in Wrt family knockdowns and knockouts. **a** Wild-type L4 animals develop one vulva on the ventral side of the animal, indicated by the single arrowhead. Scale bar is 50 μm. **b** *wrt-5(ok670)* L4 animals display a Muv phenotype where more than one ventral site undergoes vulval induction; in this example two developing vulvas are indicated with arrowheads. Scale bar 50 μm. **c** % penetrance of the Multivulva phenotype upon RNAi knockdown of a Wrt family member (x-axis) in an *ajm-1::gfp* background. *ajm-1::gfp* localises to the vulval cell apical membranes and is used to visualise the vulva using fluorescence optics. Empty vector control (L4440) animals do not display the Muv phenotype (n = 44). 0% penetrance of Muvs was recorded upon knockdown of: *wrt-1* (n = 41); *wrt-6* (n = 40); *wrt-7* (n = 39); *wrt-8* (n = 49); *wrt-9* (n = 43); *wrt-10* (n = 40). The Muv phenotype was recorded upon knockdown of *wrt-2* (n = 45); *wrt-3* (n = 41); *wrt-4* (n = 42) and *wrt-5* (n = 41). **d** % penetrance of Multivulva phenotype in wild-type (n = 40) as compared to animals carrying the *wrt-3(ok2608)* allele (n = 39) or the *wrt-5(ok670)* allele (n = 42) or the double mutant, *wrt-5(ok670);wrt-3(ok2608)* (n = 32), all in an *ajm-1::gfp* background. **e** % penetrance of Multivulva phenotype in wild-type as compared to animals carrying the *wrt-2(ok2810)* allele (n = 44) or the *wrt-4(tm1911)* allele (n = 41) or the double mutant, *wrt-4(tm1911)*;*wrt-2(ok2810)* (n = 45)*,* all in an *ajm-1::gfp* background. Black bars show mean + SEM (**c–e**). Black asterisks (****P ≤ 0.0001, ***P ≤ 0.001, **P ≤ 0.01, *P ≤ 0.05, nsP > 0.05) show statistically significant differences in the means compared to control RNAi with an unpaired *t* test (**c**) or in the means of Wrt mutants compared to WT with an unpaired *t* test (**d**, **e**)

Having observed vulval phenotypes in some Wrt family RNAi animals, we crossed in the *ajm-1::gfp* marker (which localises to apical cell membranes [30] in order to visualise and quantify these defects more precisely. RNAi knockdown of each family member revealed that members of the Wrt-3/5 clade are required for vulval fate specification. The hermaphrodite vulva Fig. 3a is a paradigm for organogenesis with a well-elucidated molecular basis underpinned by an inductive RTK-Ras-MAPK signalling cascade and subsequent lateral Notch signalling between vulval precursors [31]. Aberrant signalling can cause too many progenitors at the ventral midline to adopt a vulval cell fate giving rise to ectopic non-functional protrusions, or pseudovulvae—a phenotype known as Multivulva (Muv) Fig. 3b [32].

Members of the Warthog family have been implicated in vulval organogenesis previously [33]. Knockdown of *wrt-3* or *wrt-5* resulted in significant Muv defects (40% and 18% penetrance, respectively) whereas none of the other Wrt family members were associated with vulval defects except for the very low penetrance defects (< 5%) in *wrt-2* and *wrt-4* knockdowns (but not in *wrt-7* or *wrt-8* knockdowns) Fig. 3c. For both gene pairs that exhibited Muv phenotypes in the Wrt-3/5 and Wrt-2/4/7/8 clades, double mutants had additive but not synergistic phenotypes, again suggesting no redundancy Fig. 3d, e.

#### Members of the Wrt-1/9 clade are involved in body size regulation

We also noticed that knockdown of some Wrt family members resulted in shorter worms Table 1. Quantifying this, we observed knockdown of *wrt-1* or *wrt-9* leads to a ~ 3% decrease in body length in adult worms, whereas none of the other Wrt family members showed this significant decrease. To test for redundancy, we built a *wrt-1(tm1417),wrt-9(ok2732)* double mutant and again found no evidence of redundancy.

**Table 1:** The role of the Wrt-1/9 clade in body size regulation

Genotype	Body size 48 h post L4 (mm)	n number	*P* value^α^
Empty vector control	1.229	35	
*wrt-1*	1.213	32	< 0.0001
*wrt-2*	1.230	24	ns
*wrt-3*	1.234	28	ns
*wrt-4*	1.231	30	ns
*wrt-5*	1.231	29	ns
*wrt-6*	1.229	23	ns
*wrt-7*	1.232	26	ns
*wrt-8*	1.223	27	0.0229
*wrt-9*	1.205	33	< 0.0001
*wrt-10*	1.231	22	ns
Wild-type	1.227	30	
*wrt-1(tm1417)*	1.206	39	< 0.0001
*wrt-9(ok2732)*	1.204	40	< 0.0001
*wrt-1(tm1417);wrt-9(ok2732)*	1.204	48	ns

^α^Unpaired t tests comparing the mean body lengths between Empty Vector Control animals and Wrt gene RNAi animals; wild-type and *wrt-1(tm1417)* and *wrt-9(ok2732)* single mutants; and the *wrt-9(ok2732)* and *wrt-1(tm1417);wrt-9(ok2732)* double mutant

#### Multiple members of the Warthog family are involved in ecdysis

**Fig. 4:**
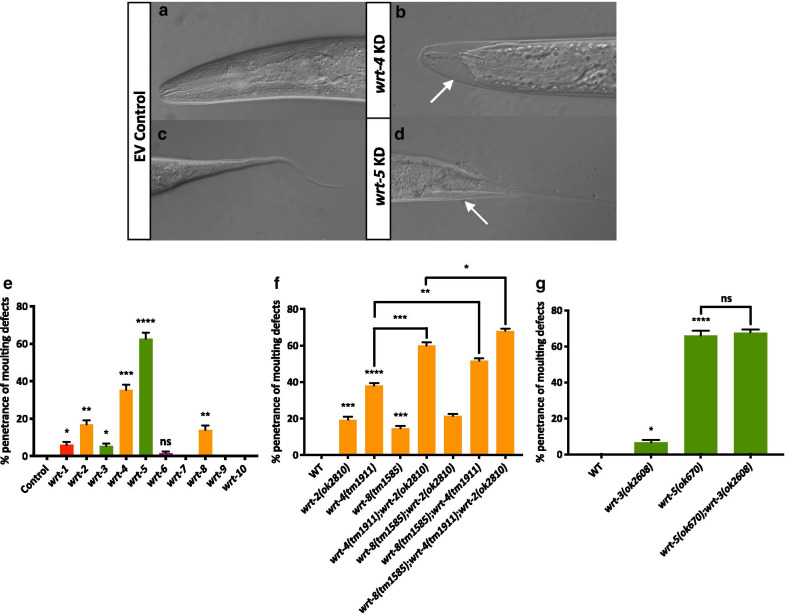
Presence of moulting and cuticle defects in Wrt family knockdowns and knockouts. **a**, **c** Wild-type worms showing head and tail, respectively. **b** *wrt-4* KD animal with an improperly shed cuticle in the head region, referred to as ‘head in a bag’ phenotype (see arrow). **d** *wrt-5* KD animal with an improperly shed cuticle in the tail region (see arrow). Scale bar 50 μm. **e** % penetrance of moulting defects present upon RNAi knockdown of a Wrt family member (x-axis). Empty vector control (L4440) animals do not display any moulting defects (n = 51). 0% penetrance of moulting defects were recorded upon knockdown of: *wrt-7* (n = 67); *wrt-9* (n = 85); *wrt-10* (n = 59). Moulting defects were recorded upon knockdown of *wrt-1* (n = 80); *wrt-2* (n = 72); *wrt-3* (n = 69); *wrt-4* (n = 80); *wrt-5* (n = 71); *wrt-6* (n = 45); *wrt-8* (n = 56). **f** % penetrance of moulting defects in wild-type (n = 50) as compared to animals carrying the *wrt-3(ok2608)* allele (n = 81) or the *wrt-5(ok670)* allele (n = 71) or the double mutant, *wrt-5(ok670);wrt-3(ok2608)* (n = 67). **g** % penetrance of moulting defects in wild-type as compared to animals carrying the *wrt-2(ok2810)* allele (n = 65), the *wrt-4(tm1911)* allele (n = 108), the *wrt-8(tm1585)* allele (n = 68) or the following double/triple mutants: *wrt-4(tm1911)*;*wrt-2(ok2810)* (n = 102); *wrt-8(tm1585)*;*wrt-2(ok2810)* (n = 87); *wrt-8(tm1585)*;*wrt-4(tm1911)* (n = 54); *wrt-8(tm1585)*;*wrt-4(tm1911)*;*wrt-2(ok2810)* (n = 103)*.* Black bars show mean + SEM (**e**–**g**). Black asterisks (****P ≤ 0.0001, ***P ≤ 0.001, **P ≤ 0.01, *P ≤ 0.05, nsP > 0.05) show statistically significant differences in the means compared to control RNAi with an unpaired *t* test (**e**) or in the means of Wrt mutants compared to WT with an unpaired *t* test (**f**, **g**)

**Fig. 5:**
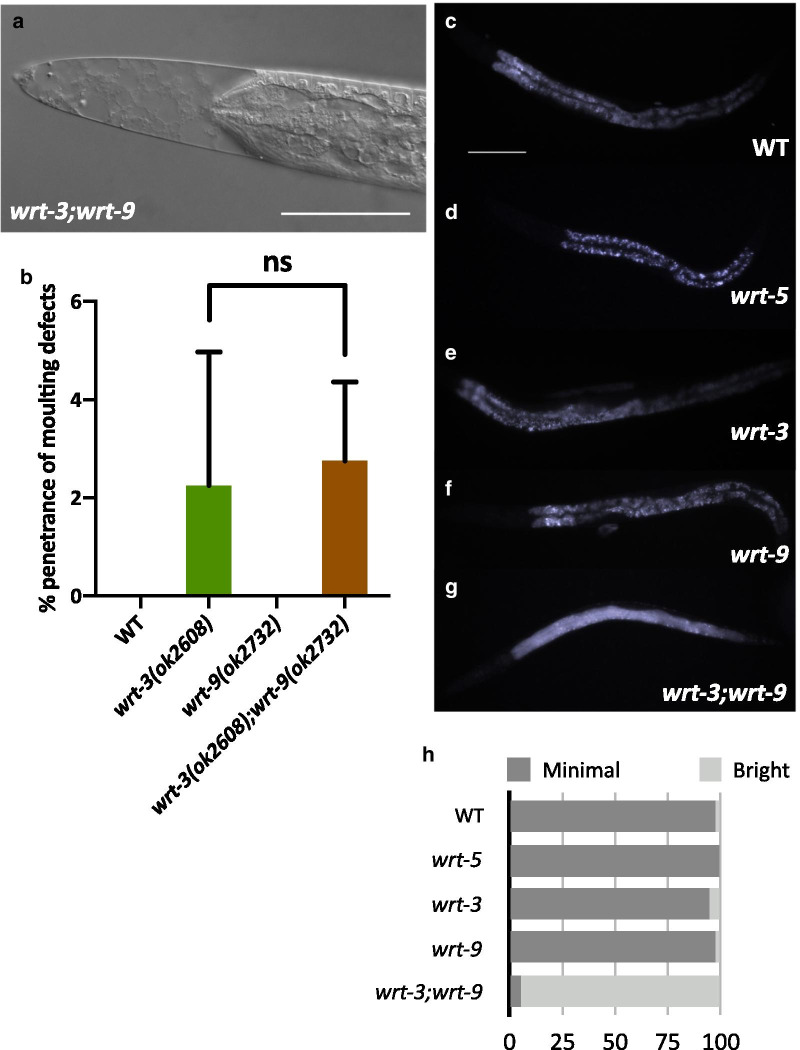
Cuticle integrity of warthog mutants. **a** Depicts the fragile and perforated cuticles of *wrt-3(ok2608);wrt-9(ok2732)* mutants that was noticed upon the initial construction of these double mutant animals. **b** Shows the % penetrance of moulting defects (+ SD) in WT (n = 53), *wrt-3(ok2608)* (n = 53), *wrt-9(ok2732)* (n = 57) and *wrt-3(ok2608);wrt-9(ok2732)* (n = 56) animals, respectively. The mean % penetrance of moulting defects present in *wrt-3(ok2608)* single mutant and *wrt-3(ok2608);wrt-9(ok2732)* double mutant animals was compared with an unpaired *t* test and found not to be significant (nsP > 0.05). **c**–**g** Depict worms which have been soaked with DAPI for 15 min and imaged using 100 ms exposure time. **h** Depicts the quantification of DAPI fluorescence using a scoring system established in [35] using this DAPI assay where the x-axis is the % of total worms imaged. **c**–**f** Represent ‘Minimal’ fluorescence, while (**g**) represents ‘Bright’ fluorescence. The fluorescence observed in (c–f) is autofluorescence, rather than DAPI stain. Wild-type (n = 45), 97.44% minimal,*wrt-5(ok670)* (n = 51), 100% minimal; *wrt-3(ok2608)* (n = 55), 94.74% minimal; *wrt-9(ok2732)* (n = 55), 97.44% minimal; *wrt-3(ok2608);wrt-9(ok2732)* (n = 59), 95.00% bright. Scale bars 50 μm

The germline, vulval and body length defects of the Wrt family mutants appear to cluster with particular clades of the phylogeny, however, we observed widespread moulting defects (exemplified in Fig. 4b, d upon knockdown of nearly all family members. Moulting is the process by which animals replace their old exoskeleton, or cuticle, with a new one [34]. The cuticle is a collagenous barrier between the animal and its external environment Fig. 4a, c. As ecdysozoans, *C. elegans* like other nematodes undergoes four moults throughout development which mark the start of each larval stage. Bürglin [14] documented the role of *wrt-5* in epidermal development and moulting as well as the cyclical expression pattern (in phase with the moulting cycle) of many Hedgehog-related genes, including the Warthogs. In light of this, we characterised the presence of moulting defects in Warthog family knockdowns Fig. 5e and found *wrt-1*, *wrt-2*, *wrt-3*, *wrt-4*, *wrt-5* and *wrt-8* all have roles in ecdysis.

While the respective penetrance of moulting defects in the single deletion mutants of *wrt-1(tm1417)*, *wrt-2(ok2810)*, *wrt-3(ok2608)*, *wrt-4(tm1911)*, *wrt-5(ok670)* and *wrt-8(tm1585)* was consistent with the RNAi knockdown data, double and triple mutant analysis showed redundancy is not exhibited between clade members with respect to moulting Fig. 4f, g. However, knockout/knockdown combinations of multiple Wrts from different clades, dubbed ‘interclade RNAi’, revealed that one pair of Wrts from different clades, *wrt-3* and *wrt-9*, are redundant for their role in cuticle biosynthesis, yet not moulting, which was later confirmed by building a *wrt-3(ok2608),wrt-9(ok2732)* double mutant Fig. 5. Upon initial imaging of these *wrt-3(ok2608),wrt-9(ok2732)* double mutant animals, it was noted that their cuticles appeared fragile and perforated Fig. 5a. Defects in the cuticle integrity of *wrt-3(ok2608),wrt-9(ok2732)* double mutants was assayed using 4′,6-diamidino-2-phenylindole (DAPI) uptake [35]. Animals in which both of the functions of these genes are abolished have a highly permeable cuticle compared to *wrt-3(ok2608)* and *wrt-9(ok2732)* single mutants, WT, or Wrt mutants that exhibit highly penetrant moulting phenotypes such as *wrt-5(ok670)* Fig. 5c–g.

It is worth noting that multiple other interclade RNAi combinations were tested over the course of this investigation, yet no additional phenotypes or non-additive effects of phenotypes already recorded were observed Additional file 8: Fig. 6).

Multiple attempts at knocking down *wrt-6* and *wrt-10* did not result in any apparent phenotypes. Recently generated putative null alleles for *wrt-6* and *wrt-10* using CRISPR/Cas9 gene editing also display no obvious gross morphological phenotype [36], and so their roles in *C. elegans* remain unknown. However, we tested if the *C. elegans* specific substitutions in *wrt-6* and *wrt-10* were driven by positive selection, as indicated by an elevated d*N*/d*S* (*ω*) ratio, but found the long divergence times were associated with saturation of d*S* and gave unreliable *ω* estimation in both cases.

## Discussion

The notion that “natural selection merely modified, while redundancy created”, has been the fundamental premise to theories of evolution by gene duplication since it was first proposed by Susumu Ohno in his seminal book in 1970 [9]. The implication that functional redundancy is simply a transient state of duplicated genes has been widely accepted in the field of evolutionary genetics, but there are instances in which redundancy is maintained between paralogue pairs for over nearly 100 million years of evolution [7, 8]. However, the pervasiveness of redundancy in large gene families has been poorly assessed. It seems intuitive that the functional redundancies in large gene families would occur exclusively between more recent duplicates, while older paralogues would have taken on neofunctionalised, non-redundant roles. To test these ideas, we characterised the duplication history and the roles of the taxon-restricted Warthog family in the nematodes.

### Reconstructing the duplication history of the Warthog family

**Fig. 6:**
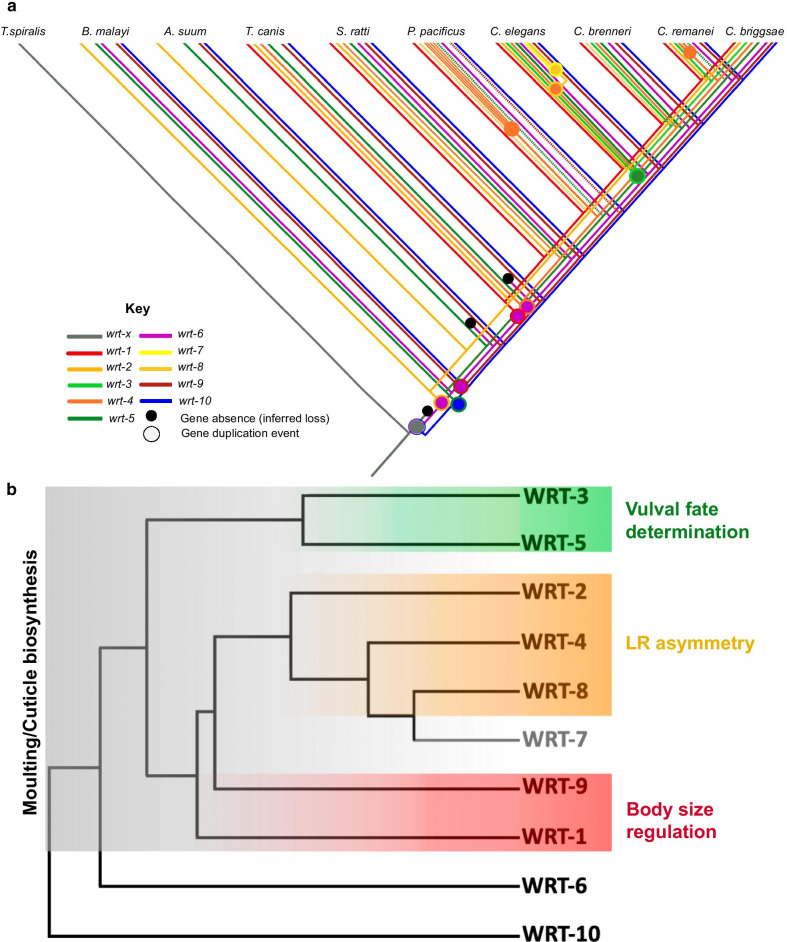
Evolution of the Warthog family. **a** Schematic detailing the duplication history of the Warthogs in the nematodes. Degenerate Wrts are represented by a dashed line. The three degenerate ‘*wrt-4*’ paralogues present in *P. pacificus* are not shown for clarity. **b** Cladogram showing the evolutionary relationships between the ten Warthogs and their functions according to the results of this investigation. *wrt-7* (grey) is a pseudogene with no obvious functionality

The extensive variation in the Warthog repertoires among nematode species as compared to the static nature of the relatively few Hedgehog genes in the bilaterians is symptomatic of the family’s vulnerability to duplication and loss. Due to the generation of high-quality genome assemblies for many species in the nematode phylum in recent years, reconstructing the duplication history of multigene families can now be done in unprecedented phylogenetic detail [37]. By combining phylogenetic, synteny and repeat sequence data, we derived the model for the duplication history of the Warthog genes as shown in Fig. 6a.

The family have likely derived from a single ancestral gene, *wrt-x*, which is still represented in *Trichinella spiralis*. This ancestral Warthog appears to have duplicated at least twice to yield a Hog-containing (wrt-1/2/4/6/7/8/9) precursor and a Hog-less precursor (wrt-3/5/10) less than 400 mya. These two progenitors presumably then expanded with the radiation of Chromadorea to create a complement of five Warthogs (*wrt-2*, *wrt-5*, *wrt-6*, *wrt-9*, *wrt-10*) which are represented in nearly all the extant Clade III nematodes studied in this investigation, with the exception of the independent loss of *wrt-6* in *A. suum*. Following their generation by tandem duplication, *wrt-2* and *wrt-9* subsequently lost their Hog domains. The Hog-containing progenitor is envisaged to have given rise to *wrt-1* and *wrt-4* in *T. canis* and other lineages (Clade IV and V nematodes), as well as *wrt-7* and *wrt-8* in *C. elegans* less than 10 mya [37]. The Hog-less wrt-3/5 precursor subsequently duplicated to yield *wrt-3* in the *Caenorhabditis* genus less than 100 mya.

### Neofunctionalisation of Warthog family genes reflects cladistic architecture

Because of the genetic tractability of *C. elegans*, evolutionary hypotheses derived from the duplication history of large multigene families can be tested using robust genetic techniques. We sought to test the relationship between the age of gene duplicates and the likelihood of functional redundancy in the Warthog family. Overall, we found hitherto unreported roles for the Warthog family in the generation of middle body LR asymmetry (*wrt-2*, *wrt-4* and *wrt-8*), cell fate specification in the developing vulva (*wrt-3* and *wrt-5*), and body size regulation (*wrt-1* and *wrt-9*). These roles associate strongly with particular clades of the Warthog phylogeny Fig. 6b. Thus, we conclude that these clades have neofunctionalised in aspects of post-embryonic development.

Surprisingly, we did not find any instances of complete functional redundancy between family members in these neofunctionalised clades, implying they operate in different pathways. If the Warthogs encode ligands that operate in different signalling pathways, this would explain the additivity of the phenotypes observed in the mutants of even closely related Wrt genes, such as *wrt-2*, *wrt-4* and *wrt-8*; *wrt-3* and *wrt-5*; and *wrt-1* and *wrt-9*. This implies a highly robust network of genes involved in these developmental processes.

*wrt-3* is a recently derived Warthog, only found in members of the *Caenorhabditis* genus which arose less than 100 mya. In light of this, the severe and highly penetrant phenotypes that it exhibits are unexpected. Thus, it can be stated that recently duplicated Warthogs are not only non-redundant with one another, but in the instance of *wrt-3*, have also assumed critical developmental roles including in organogenesis. As one of the more recently derived members of the Wrt-2/4/7/8 clade, *wrt-7* appears to have completely pseudogenised, having no obvious functionality or expression pattern throughout development [26].

### The roles and redundancies of the Warthog family in ecdysis

Throughout this investigation, we observed many moulting defects in Warthog family mutants. As such, we systematically characterised the role of each member in moulting and found that *wrt-1*, *wrt-2*, *wrt-3*, *wrt-4*, *wrt-5* and *wrt-8* are all involved to some extent in this process. The role of some Warthog family members in moulting and the oscillatory expression patterns of several Warthogs has implicated the family in ecdysis in previous studies [38, 39]. As many other members of the Hedgehog-related (Hh-r) [26, 39, 40] and Patched/Patched-related (Ptc-r) superfamilies are involved in moulting (reviewed by [34]), we propose that ecdysis is the ancestral role of the divergent ‘Hedgehog’ pathway in Nematoda and that Hh-r and Ptc-r genes were at least ancestrally in the same pathway.

We did not find any instances of redundancy in the Warthog family with respect to moulting, either between those in the same clade or those in different clades. The only instance of functional redundancy observed in this investigation is between Warthogs in different clades, *wrt-3* and *wrt-9,* in cuticle biosynthesis, but not moulting. We propose that these surprising patterns of redundancy are the consequence of paralogue specialisation following gene duplication. It is likely that the ancestor of the Warthog family was a pleiotropic regulator of ecdysis, involved in both shedding the old cuticle and synthesising the new, yet following the generation of the ten members by many tandem gene duplication events, these functions were distributed among paralogues such that *wrt-1, wrt-2, wrt-4, wrt-5* and *wrt-8* all retained moderate roles in moulting, while *wrt-3* and *wrt-9* have independently specialised in cuticle biosynthesis.

### Paralogy relationships do not predict redundancy relationships in the Warthog family

The unexpected redundancy relationship between *wrt-3* and *wrt-9* could be explained by their independent specialisation in cuticle biogenesis, giving rise to a rarely described phenomenon of stable redundancy (SR) preserved through unexpectedly long evolutionary timescales. This contrasts with patterns of redundancy often observed between many recently derived paralogous genes, which we term ‘transient-duplication-associated-redundancy’ (TDAR). TDAR can be thought of as the evolutionarily unstable short term consequence of duplicated genes, which inevitably exists immediately following a gene duplication event prior to a period of divergence. SR on the other hand, is a possible means by which gene duplications could instil robustness in gene regulatory networks, and thus provides a long term selective advantage which allows it to persist over long evolutionary timescales.

## Methods

### Strains and maintenance

All *C. elegans* strains described were derived from the N2 Bristol wild-type strain. Worms were maintained in a temperature-controlled laboratory at 20 °C. All maintenance and manipulation of strains was performed as previously described [41]. The details of the deletion alleles used in this study are provided in Additional file 11: Table 4. *wrt-1(tm1417)*, *wrt-4(tm1911)*, and *wrt-8(tm1585)* were isolated by the National BioResource Project http://www.shigen.nig.ac.jp/c.elegans/index.jsp. *wrt-2(ok2810)*, *wrt-3(ok2608)*, *wrt-5(ok670)*, *wrt-7(ok3271)* and *wrt-9(ok2732)* were obtained from the Caenorhabditis Genetics Center, University of Minnesota http://www.cgc.umn.edu.

Strains used in this study are listed in Additional file 11: Table 4. All strains were outcrossed a minimum of four times prior to performing genetic crosses and phenotypic characterisation. All strains used in this investigation were obtained by the performance of genetic crosses and all alleles (excluding *him-5(e1490)* and *ajm-1::gfp*) were followed throughout via genotyping PCR. A complete list of the primers used in this investigation can be found in Additional file 12: 11.

### Microscopy and phenotypic characterisation

All microscopy and phenotypic characterisation was carried out at room temperature. For light and fluorescence microscopy, animals were mounted on 2% agarose pads. Worms were picked into 3 μl of 20 mM levamisole (anaesthetic) and covered with a coverslip. Worms were visualised with an epifluorescent Zeiss microscope fitted with Nomarski (DIC), GFP and DAPI filters and a 63 × oil immersion objective and Axiovision software was used to capture fluorescent and DIC images.

The intestinal twist in 1.5-fold embryos was scored using Nomarski microscopy. Only embryos that had a lateral presentation such that the rectum was present in the same focal plane as the intestine were analysed, as described in Hermann et al. [42]. Hermaphrodite gut/gonad orientation was scored with L4 worms in the ventral view (lefthand plane), as previously described in Alcorn et al. [43], and imaged at 40 × or 63 × magnification.

For body size quantification, hermaphrodite animals were picked at the L4 stage and photographed as young adults 48 h later. Body size was measured manually using Axiovision software. L4 stage animals/early adults were used to score the Multivulva and moulting defect phenotypes.

### RNA interference (RNAi)

RNAi was provided by feeding [44]. All RNAi constructs were obtained from the commercially available Source Bioscience Ahringer whole-genome library [45] and were validated by sequencing prior to all experiments.

### Cuticle permeability assays

Cuticle permeability to 4′,6-diamidino-2-phenylindole (DAPI) was assayed as described [35]. In brief, L4 larvae were washed from plates with M9 buffer prior to staining with DAPI (5 μg/ml each in M9 buffer) for 15 min at room temperature with gentle agitation. Subsequently, worms were washed three times with M9 buffer, followed by fluorescence imaging. For microscopy, worms were mounted onto 2% agarose pads, anaesthetised with 3 μl of 20 mM levamisole and sealed with a coverslip before imaging on a Zeiss Axioplan 2 microscope. Samples were observed with a Zeiss Plan Neofluar 20×/0.50 Ph2 objective, images captured using a Zeiss AxioCam and the software AxioVision 4.8. DAPI accumulation was imaged at 100msec exposure time.

### Molecular phylogenetic and other genomic analysis

*Caenorhabditis elegans* Wrt sequences were obtained from WormBase (http://wormbase.org) and (PSI-)BLAST searched [46] against the genomes of selected nematode species (using the web service default parameters). We selected representatives from the phylum Nematoda on the basis of genome quality and completeness. Consequently, the sequences are either from major parasites, including: *Trichinella spiralis*, *Brugia malayi*, *Ascaris suum* and *Toxocara canis*, or model organisms, including: *Pristionchus pacificus*, *Caenorhabditis briggsae*, *Caenorhabditis remanei*, *Caenorhabditis brenneri*. Multiple sequence alignments were carried out using SeaView software version 4.6.2 [47] and the CLUSTAL Omega programme (default parameters) was used to locally improve the alignment, which was further adjusted by eye. Phylogenetic tree construction was achieved using the Bayesian algorithm in MrBayes version 3.2 [48]. Bayesian inference was performed using the Markov chain Monte Carlo method. Two independent Markov chains were run, each with 1 million iterations with default heating parameters. The first 25% of the trees were discarded as burn-in before compiling consensus trees and summary statistics. Posterior probabilities at each internal node were taken as a measure of support. The maximum likelihood phylogeny was constructed using IQ-TREE [49] and its built-in ModelFinder software [50]. Branch support was calculated running 10,000 replicates of the SH-like approximate likelihood ratio test and ultrafast bootstrap (10,000 replicates). Both tree figures were rendered with FigTree (http://tree.bio.ed.ac.uk/software/figtree/).

To map synteny and paralogy relationships, genes directly adjacent to the Wrt loci in *C. elegans* were searched by tBLASTn of their predicted proteins across the other genomes. If an orthologue was present/detected, their genomic location in a given species was compared to the position of the orthologue in the *C. elegans* reference genome.

RepeatMasker [51] was used to screen for repetitive sequences and regions of low complexity using default parameters in all ten *C. elegans* Warthogs.

### Testing for positive selection using phylogenetic analysis by maximum likelihood (PAML)

We tested for positive selection using CodeML implemented in PAML [52], using a branch-model to estimate the d*N*/d*S* ratio by assigning two independent ratios, specifying the branch leading to *C. elegans* (model = 2, NSsites = 0). We ran two analyses (one testing for positive selection in the *wrt-6* clade and another testing for positive selection in the *wrt-10* clade) and compared the null model (M0) to a two-ratio branch model (specifying the *C. elegans* lineage as foreground in both).

## Supplementary Information

**Additional file 1: Fig. 1.** Multiple sequence alignment of all the Wart domain proteins mined from the predicted proteomes of various nematode species (Species abbreviations: *Tnat, Trichinella nativa*; Tmur, *Trichinella murelli*; TspT6, *Trichinella sp. T6*; TspT8, *Trichinella sp. T8*; TspT9, *Trichinella sp. T9*; Tpap, *Trichinella papuae*; Tpat, *Trichinella patagoniensis*; Tnel, *Trichinella nelsoni*; Tpseudo, *Trichinella pseudospiralis*; Trchrs_su, *Trichuris suis;* Ts, *Trichinella spiralis*; Bm, *Brugia malayi*; As, *Ascaris suum*; Tc, *Toxocara canis*; Sr, *Strongyloides ratti*; Pp, *Pristionchus pacificus*; Cbre, *Caenorhabditis brenneri*; Cbri, *C. briggsae*; Cr, *C. remanei*; Ce, *C. elegans*.). Alignment was constructed using SeaView software version 4.6.2 and the CLUSTAL Omega programme (default parameters) was used to locally improve the alignment, which was further refined by eye. **Fig. 2.** IQ-TREE maximum likelihood molecular phylogenetic analysis of the Wart domain sequences mined from selected nematode genomes (Species abbreviations: *Tnat, Trichinella nativa*; Tmur, *Trichinella murelli*; TspT6, *Trichinella sp. T6*; TspT8, *Trichinella sp. T8*; TspT9, *Trichinella sp. T9*; Tpap, *Trichinella papuae*; Tpat, *Trichinella patagoniensis*; Tnel, *Trichinella nelsoni*; Tpseudo, *Trichinella pseudospiralis*; Trchrs_su, *Trichuris suis;* Ts, *Trichinella spiralis*; Bm, *Brugia malayi*; As, *Ascaris suum*; Tc, *Toxocara canis*; Sr, *Strongyloides ratti*; Pp, *Pristionchus pacificus*; Cbre, *Caenorhabditis brenneri*; Cbri, *C. briggsae*; Cr, *C. remanei*; Ce, *C. elegans*.). The node labels are ultrafast bootstrap support values. The tree was generated in FigTree. **Table 1. (1.1)** Table lists the accession numbers of the additional (degenerate) Wart domain containing sequences mined from the nematode species in this investigation. Where legitimate Wart domain containing genes are included, these have been named by our investigation according to our findings, i.e. the previously unannotated four *wrt-4* paralogues in *P. pacificus* and the *wrt-x* orthologues and putative Hh homologues in Clade I species. **(1.2)** Table lists the species used in this investigation and the NCBI BioProject accession number of the edition of their genome used. **Fig. 3.** Alignment of ‘*wrt-4′* paralogues in *P. pacificus* using Clustal Omega (automatic parameters). Only III_PDM72491.1 (Pp_WRT-4iii, chromosome X) is predicted to encode a Hog/Hint motif, i.e. an intein-mediated protein splicing domain (GO0016539) and a protein autoprocessing domain (GO0016540) as annotated by InterProScan 5 [61]. **Table 2.** Table details the genes directly adjacent to each Warthog family orthologue in a particular nematode species. Purple font indicates that the adjacent gene is not orthologous to the adjacent gene beside the orthologous Warthog in *C. elegans*. Green font indicates that the adjacent gene is orthologous to the adjacent gene beside the orthologous Warthog in *C. elegans*. ‘-’ indicates that there was a ‘gene desert’ adjacent to the Warthog locus or that the contig finished and so syntenic relationships could not be established. ‘Gene deserts’ are arbitrarily defined here as regions on a chromosome that do not feature any open reading frames for over 3.5 kb. All genes are represented with their WormBase/NCBI accession numbers. **Fig. 4. (A)** *wrt-7* RNAi knockdown performed on different genotypes. From the left, empty vector control RNAi performed on wildtype, *wrt-4(tm1911),wrt-2(ok2810)*, *wrt-8(ok1585);wrt-2(ok2810)*, *wrt-8(ok1585);wrt-4(tm1911)*, *wrt-8(ok1585);wrt-4(tm1911);wrt-2(ok2810)* and *rrf-3(pk1426)* animals; and *wrt-7* RNAi performed on wildtype, *wrt-4(tm1911);wrt-2(ok2810)*, *wrt-8(ok1585);wrt-2(ok2810)*, *wrt-8(ok1585);wrt-4(tm1911)*, *wrt-8(ok1585);wrt-4(tm1911);wrt-2(ok2810)* and *rrf-3(pk1426)* animals. **(B)** Wrt-2/4/8 knockdowns on WT and *wrt-7(ok3271)* animals. From the left, empty vector control RNAi performed on wildtype and *wrt-7(ok3271)* mutants; *wrt-2* RNAi performed on wildtype and *wrt-7(ok3271)* mutants; *wrt-4* RNAi performed on wildtype and *wrt-7(ok3271)* mutants; and *wrt-8* RNAi performed on wildtype and *wrt-7(ok3271)* mutants. Black bars show mean + SEM. All comparisons are not significant. **Table 3.** Table details the naturally occurring variations found in wild isolates of *C. elegans* in the open reading frame of *wrt-7* as retrieved from the CeNDR database (Cook et al. 2016). The precise mutational change (and corresponding amino acid change) is listed according to the reference (Bristol N2) and the corresponding change and the effect on the predicted protein is given in the eighth and ninth columns, respectively. **Fig. 5.** *wrt-3(ok2810)* animals show severe morphological defects in the middle body despite being outcrossed six times. *wrt-3* RNAi animals also display the same middle body defects. The gonad arms appear both abnormally shaped and fragmented, often miniaturised. Large vacuoles also distort the middle body anatomy. The vulvae (arrowheads) also protrude. The severity of the morphological defects meant that we were unable to observe the left–right asymmetry of the middle body in *wrt-3(ok2810)* mutant animals. Scale bar = 50 μm. **Fig. 6. (A)** % penetrance of LR defects recorded on specific Wrt family RNAi knockdown on *wrt-8(tm1585);wrt-4(tm1911);wrt-2(ok2810)* triple mutants (Empty Vector Control, n = 20; *wrt-1*, n = 23; *wrt-5*, n = 20; *wrt-6*, n = 19; *wrt-9*, n = 25; *wrt-10*, n = 20). *wrt-3* knockdown was not performed due to the severe middle body morphological defects it gives rise to (SD8) **(B)** % penetrance of Muv defects recorded on specific Wrt family RNAi knockdown on *wrt-5(ok670);wrt-3(ok2608)* double mutants. (Empty Vector Control, n = 19; *wrt-1*, n = 19; *wrt-2*, n = 21; *wrt-4*, n = 24; *wrt-6*, n = 22; *wrt-7*, n = 26; *wrt-8*, n = 20; *wrt-9*, n = 29; *wrt-10*, n = 20) **(C)** Body size (48 h post L4) of *wrt-3(ok2608);wrt-9(ok2732)* double mutants upon knockdown of specific Wrt family members. Black bars show mean + SEM. Black asterisks show *P ≤ 0.05, all other comparisons are not statistically significant, i.e. P > 0.05. **Fig. 7. (A)** Gene tree of Warthog family in selected nematode species as shown in Fig. 6 of the manuscript. **(B)** *wrt-x* gene tree (grey). **(C)** *wrt-1* gene tree (bright red). **(D)** *wrt-2* gene tree (bright orange). **(E)** *wrt-3* gene tree (bright green). **(F)** *wrt-4* gene tree (dark orange). **(G)** *wrt-5* gene tree (dark green). **(H)** *wrt-6* gene tree (purple). **(I)** *wrt-7* gene tree (yellow). **(J)** *wrt-8* gene tree (sunset orange). **(K)** *wrt-9* gene tree (dark red). **(L)** *wrt-10* gene tree (blue). **Table 4.** Table details the strains used in this investigation. All strains were derived from Bristol N2 worms. Warthog mutants were outcrossed with CB4088 a minimum of four times (and in the case of RB2125 and VC2083, six times) prior to use, i.e. phenotypic characterisation and the performance of genetic crosses. All genotypes in cross progeny were followed throughout by PCR except strains crossed into P57657.

## Abbreviations

*ajm-1:gfp*: Apical junction marker-1 (green fluorescent protein)
CRISPR/Cas9: Clustered regularly interspaced short palindromic repeats
DDC: Duplication-degeneration-complementation
Dhh: Desert Hedgehog
*glp-1*: Abnormal GermLine Proliferation
GRD: GRounDhog
GRL: Ground-Like
Hh-r: Hedgehog-related
Ihh: Indian Hedgehog
*lin-12*: LINeage defective -12
LR: Left–right
Muv: Multivulva phenotype
MYA: Million years ago
PAML: Phylogenetic analysis by maximum likelihood
Pt-r: Patched-related
QUA: QUAhog
RNAi: RNA interference
*rrf-3(pk1426)*: RNA-dependent RNA polymerase family
RTK-Ras-MAPK: Receptor-tyrosine-kinase/mitogen-activated-protein-kinase
Shh: Sonic Hedgehog
SR: Stable redundancy
TDAR: Transient duplication associated redundancy
WRT (wrt): WaRThog
WT: Wild-type

## Availability of data and material

All data generated or analysed during this study are included in this published article (and its supplementary information files) or are available from the corresponding author on reasonable request. Worm strains generated for the purposes of this study are also available from the corresponding author on reasonable request.
