# Supplementary material for: Extensive non-redundancy in a recently duplicated developmental gene family
Source: BMC Ecol Evol. 2021 Mar 1;21:33. doi: 10.1186/s12862-020-01735-z (PMC7919330; doi:10.1186/s12862-020-01735-z)
Supplement: Supplementary file 1 — Additional file 1: Fig. 1. Multiple sequence alignment of all the Wart domain proteins mined from the predicted proteomes of various nematode species (Species abbreviations: Tnat, Trichinella nativa; Tmur, Trichinella murelli; TspT6, Trichinella sp. T6; TspT8, Trichinella sp. T8; TspT9, Trichinella sp. T9; Tpap, Trichinella papuae; Tpat, Trichinella patagoniensis; Tnel, Trichinella nelsoni; Tpseudo, Trichinella pseudospiralis; Trchrs_su, Trichuris suis; Ts, Trichinella spiralis; Bm, Brugia malayi; As, Ascaris suum; Tc, Toxocara canis; Sr, Strongyloides ratti; Pp, Pristionchus pacificus; Cbre, Caenorhabditis brenneri; Cbri, C. briggsae; Cr, C. remanei; Ce, C. elegans.). Alignment was constructed using SeaView software version 4.6.2 and the CLUSTAL Omega programme (default parameters) was used to locally improve the alignment, which was further refined by eye. Fig. 2. IQ-TREE maximum likelihood molecular phylogenetic analysis of the Wart domain sequences mined from selected nematode genomes (Species abbreviations: Tnat, Trichinella nativa; Tmur, Trichinella murelli; TspT6, Trichinella sp. T6; TspT8, Trichinella sp. T8; TspT9, Trichinella sp. T9; Tpap, Trichinella papuae; Tpat, Trichinella patagoniensis; Tnel, Trichinella nelsoni; Tpseudo, Trichinella pseudospiralis; Trchrs_su, Trichuris suis; Ts, Trichinella spiralis; Bm, Brugia malayi; As, Ascaris suum; Tc, Toxocara canis; Sr, Strongyloides ratti; Pp, Pristionchus pacificus; Cbre, Caenorhabditis brenneri; Cbri, C. briggsae; Cr, C. remanei; Ce, C. elegans.). The node labels are ultrafast bootstrap support values. The tree was generated in FigTree. Table 1. (1.1) Table lists the accession numbers of the additional (degenerate) Wart domain containing sequences mined from the nematode species in this investigation. Where legitimate Wart domain containing genes are included, these have been named by our investigation according to our findings, i.e. the previously unannotated four wrt-4 paralogues in P. pacificus and the w [file 12862_2020_1735_MOESM1_ESM.docx]

Supplementary material for:

**Extensive Non-redundancy in a Recently Duplicated Developmental Gene Family**

Emily A. Baker, Sophie P.R. Gilbert, Sebastian M. Shimeld & Alison Woollard

Content:

Additional File 1: **Wart domain alignment**

Additional File 2: **Maximum Likelihood IQ-TREE phylogenetic analysis of nematode Wart domains**

Additional File 3: **Updated Warthog nomenclature**

Additional File 4: ***wrt-4* complement in *Pristionchus pacificus***

Additional File 5: **Synteny analysis**

Additional File 6: ***wrt-7* is nonfunctional in *C. elegans***

Additional File 7: **Analysing the highly polymorphic *wrt-7* alleles in *C. elegans* wild isolates**

Additional File 8: **Severe morphological defects in *wrt-3* defective animals**

Additional File 9: **Interclade RNAi reveals no further redundancy in the Warthog family**

Additional File 10: **Deconstruction of Figure 6**

Additional File 11: **Strains and deletion alleles used in this investigation**

Additional File 12: **Oligonucleotides used in this investigation**

**Additional File 1: Wart domain alignment**


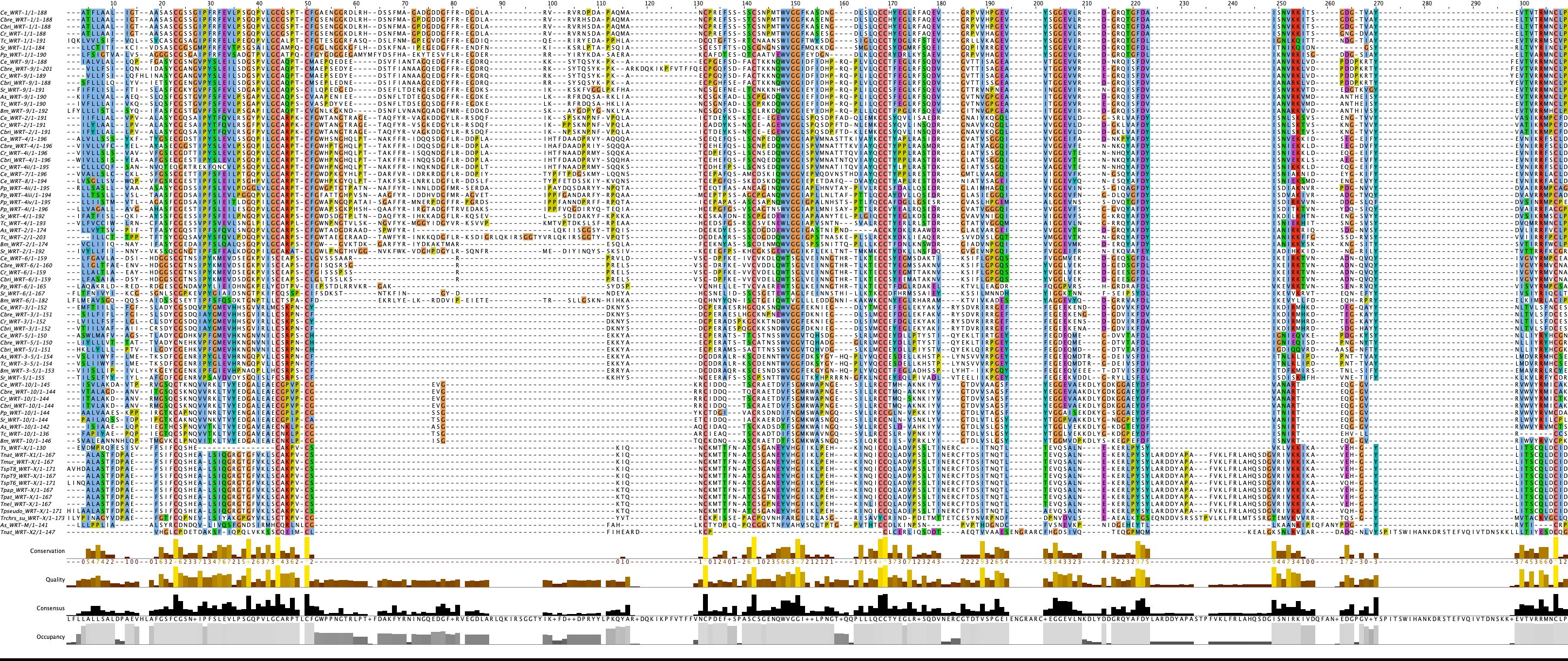


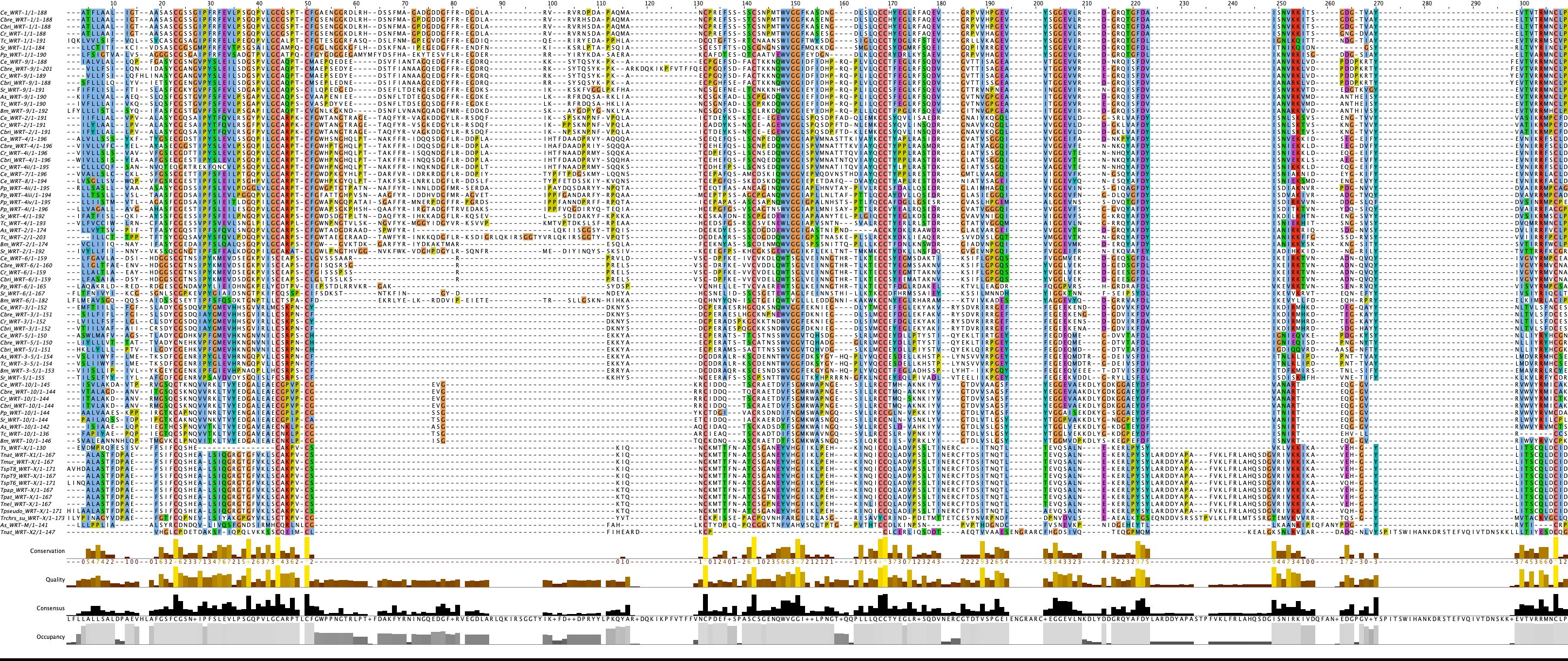


**Additional File 2: Maximum likelihood IQ-TREE phylogenetic analysis of nematode Wart domains**

**Additional File 3: Updated Warthog nomenclature**

| **1.1** | **Warthog (Wrt)/Hedgehog (Hh) identity** | **Accession number** |
| --- | --- | --- |
| ***Trichuris trichiura*** | ***hh-1*** | **CDW56653.1** |
| ***Trichuris suis*** | ***wrt-x*** | **KFD66751.1** |
|  | ***hh-1*** | **KFD51835.1** |
| ***Soboliphyme baturini*** | ***hh-1*** | **VDO93373.1** |
|  | ***hh-2*** | **VDO93372.1** |
| ***Trichinella nativa*** | ***wrt-x.1*** | **KRZ55369.1** |
|  | ***wrt-x.2*** | **KRZ61985.1** |
|  | ***hh-1*** | **KRZ52959.1** |
|  | ***hh-2*** | **OUC48110.1** |
|  | ***hh-3*** | **OUC48109.1** |
| ***Trichinella nelsoni*** | ***wrt-x*** | **KRX17290.1** |
|  | ***hh-1*** | **KRX14913.1** |
| ***Trichinella sp. T6*** | ***wrt-x*** | **KRX84097.1** |
|  | ***hh-1*** | **KRX81561.1** |
| ***Trichinella sp. T8*** | ***wrt-x*** | **KRZ95519.1** |
|  | ***hh-1*** | **KRZ90764.1** |
| ***Trichinella sp. T9*** | ***wrt-x*** | **KRX56392.1** |
|  | ***hh-1*** | **KRX55007.1** |
| ***Trichinella pseudospiralis*** | ***wrt-x*** | **KRZ25577.1** |
|  | ***hh-1*** | **KRY83366.1** |
| ***Trichinella zimbabwensis*** | ***hh-1*** | **KRZ08543.1** |
| ***Trichinella patagoniensis*** | ***wrt-x*** | **KRY09542.1** |
|  | ***hh-1*** | **KRY13248.1** |
| ***Trichinella murelli*** | ***wrt-x*** | **KRX43845.1** |
|  | ***hh-1*** | **KRX39880.1** |
| ***Trichinella papue*** | ***wrt-x*** | **KRZ73920.1** |
|  | ***hh-1*** | **KRZ71040.1** |
| ***Trichinella spiralis*** | ***wrt-x*** | **KRY35028.1** |
|  | ***hh-1*** | **ABO15010.1** |
|  | ***hh-2*** | **KRY29053.1** |
| ***Pristionchus pacificus*** | ***wrt-2 -*like*** | **PDM64744.1** |
|  | ***wrt-4i*** | **PDM79116.1** |
|  | ***wrt-4ii*** | **PDM65807.1** |
|  | ***wrt-4iii*** | **PDM72491.1** |
|  | ***wrt-4iv*** | **PDM69003.1** |
|  | ***wrt-4 -*like*** | **PDM60764.1** |
|  | ***wrt-4 -*like*** | **PDM75978.1** |
|  | ***wrt-4 -*like*** | **PDM80143.1** |
|  | ***wrt-5 -*like*** | **KKA76232.1** |
|  | ***wrt-9 -*like*** | **PDM64744.1** |
| ***Caenorhabditis brenneri*** | ***wrt-2 -*like*** | **EGT29941.1** |
| ***Caenorhabditis remanei*** | ***wrt-5 -*like*** | **XP_003096339.1** |
|  | ***wrt-2* -like*** | **OZG06368.1** |

| **1.2. Species** | **Details of genome assembly used in this investigation (NCBI BioProject, publication, etc.)** |
| --- | --- |
| *Ascaris suum* | PRJNA62057, Jex et al. (2011) |
| *Brugia malayi* | PRJNA10729, Ghedin et al. (2007) |
| *Caenorhabditis brenneri* | PRJNA20035 |
| *Caenorhabditis briggsae* | PRJNA509247, Stein et al. (2003) |
| *Caenorhabditis elegans* | PRJNA13758, *C. elegans* Sequencing Consortium (1998) |
| *Caenorhabditis remanei* | PRJNA248909, Fierst et al. (2015) |
| *Pristionchus pacificus* | PRJNA12644, Dieterich et al. (2008) |
| *Soboliphyme baturini* | PRJEB516 |
| *Strongyloides ratti* | PRJNA304930, Foth et al. (2014) |
| *Toxocara canis* | PRJNA248777, Zhu et al. (2015) |
| *Trichinella murelli* | PRJNA257433, JYDJ01000109.1, Korhonen et al. (2016) |
| *Trichinella nativa* | PRJNA179527, Korhonen et al. (2016) |
| *Trichinella nelsoni* | PRJNA257433, JYDL01000090.1, Korhonen et al. (2016) |
| *Trichinella papuae* | PRJNA257433, JYDO01000057.1, Korhonen et al. (2016) |
| *Trichinella patagoniensis* | PRJNA257433, JYDO01000269.1, Korhonen et al. (2016) |
| *Trichinella pseudospiralis* | PRJNA257433, JYDO01000099.1, Korhonen et al. (2016) |
| *Trichinella species T6* | PRJNA257433, JYDO01000008.1, Korhonen et al. (2016) |
| *Trichinella species T8* | PRJNA257433, JYDO010000012.1, Korhonen et al. (2016) |
| *Trichinella species T9* | PRJNA257433, JYDO01000124.1, Korhonen et al. (2016) |
| *Trichinella spiralis* | PRJNA257433, JYDO010000058.1, Korhonen et al. (2016) |
| *Trichinella zimbabwensis* | PRJNA257433, JYDO01000085.1, Korhonen et al. (2016) |
| *Trichuris suis* | PRJNA179528, Korhonen et al. (2016) |
| *Trichuris trichiura* | PRJEB535, Foth et al. (2014) |

**Additional File 4: *wrt-4* complement in *Pristionchus pacificus***


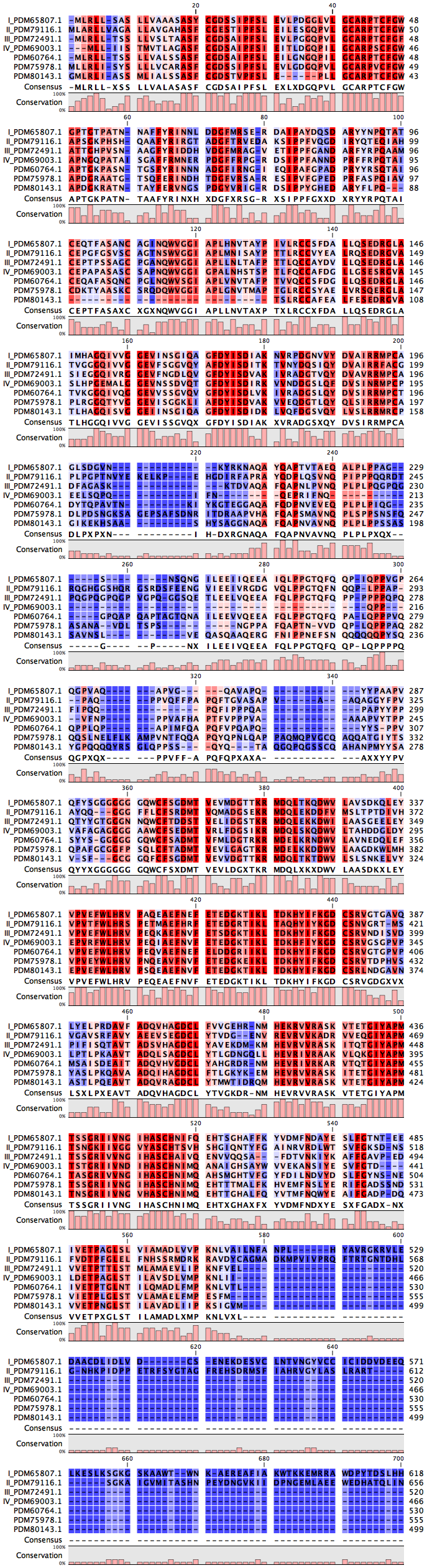


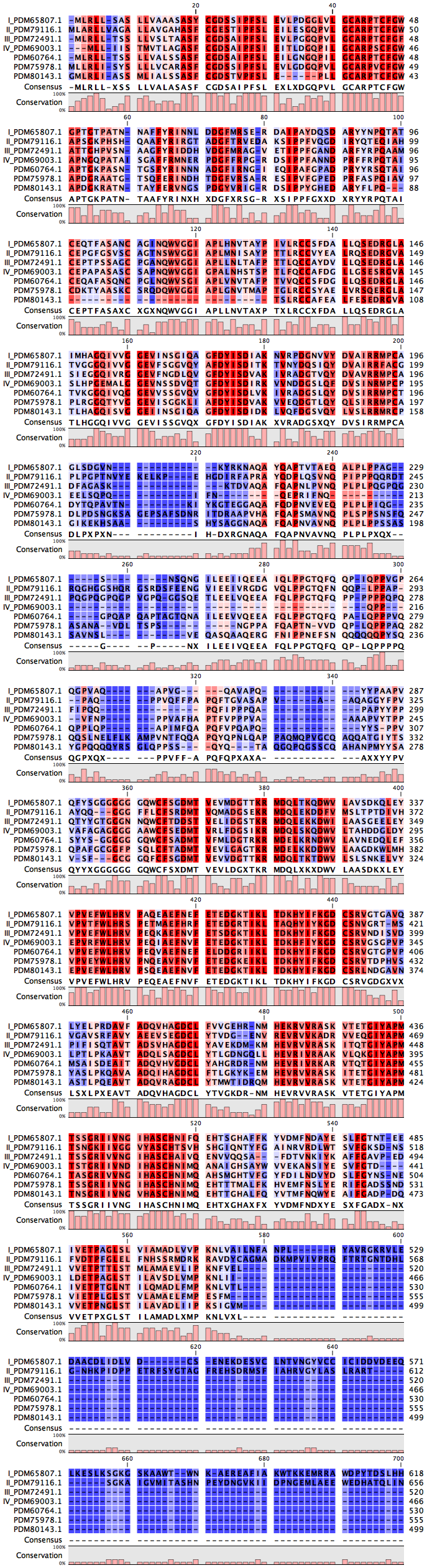


**Additional File 5: Synteny Analysis**

| **Species** | **5’ Neighbour** | **Warthog orthologue** | **3’ Neighbour** | **Comment** |
| --- | --- | --- | --- | --- |
| ***C. elegans*** | **ZK1290.5.1** | ***wrt-1*** | ***wrt-10*** |  |
|  | ***pccb-1*** | ***wrt-2*** | ***sec-3*** |  |
|  | **F38E11.6** | ***wrt-3*** | ***cng-3*** |  |
|  | ***srxa-9*** | ***wrt-4*** | ***bus-17*** |  |
|  | ***pcn-1*** | ***wrt-5*** | ***ilys-6*** |  |
|  | ***sax-3*** | ***wrt-6*** | **C12D12.3** |  |
|  | ***srw-113*** | ***wrt-7*** | ***clec-228*** | **Loci (adjacent to one another) map to *Cbri_wrt-4* (*C. briggsae*)** |
|  | ***clec-229*** | ***wrt-8*** | **C29F3.3** |  |
|  | **H02F09.2** | ***wrt-9*** | **H11E01.3** |  |
|  | ***wrt-1*** | ***wrt-10*** | **ZK1290.13** |  |
| ***C. brenneri*** | **Cbn12985** | ***wrt-1 (CBN24485)*** | ***wrt-10*** |  |
|  | **Cbn-*pccb-1*** | ***wrt-2 (CBN13100)*** | **Cbn-*sec-3*** | ***Degenerate (multiple cysteines missing)*** |
|  | **Cbn25685** | ***wrt-3 (CBN4088)*** | **Cbn31929** |  |
|  | **Cbn-*srxa-9*** | ***wrt-4 (CBN00608)*** | **Cbn-*bus-17*** |  |
|  | **Cbn-*pcn-1*** | ***wrt-5 (CBN18301)*** | **Cbn08017** |  |
|  | **Cbn-*sax-3*** | ***wrt-6 (CBN23203)*** | **Cbn16646** |  |
|  | **Cbn22677** | ***wrt-9 (CBN13835)*** | **-** |  |
|  | ***wrt-1*** | ***wrt-10 (CBN25699)*** | **Cbn13535** |  |
| ***C. remanei*** | **Cre26393** | ***wrt-1 (CRE26395)*** | **Cre26108** |  |
|  | **Cre-*pccb-1*** | ***wrt-2 (CRE00439)*** | **Cre-*sec-3*** |  |
|  | **Cre03559** | ***wrt-3 (CRE03560)*** | **Cre-*cng-3*** |  |
|  | **Cre-*srxa-9*** | ***wrt-4 (CRE19116)*** | **Cre-*bus-17*** |  |
|  | **Cre-*pcn-1*** | ***wrt-5 (CRE13565)*** | **-** | ***Degenerate (multiple cysteines missing)*** |
|  | **Cre-*sax-3*** | ***wrt-6 (CRE00892)*** | **Cre00890** |  |
|  | **Cre24213** | ***wrt-9 (CRE24214)*** | **Cre24215** |  |
|  | ***wrt-1*** | ***wrt-10 (CRE26108)*** | **Cre26394** |  |
| ***C. briggsae*** | **Cbg12985** | ***wrt-1 (CBG12986)*** | ***wrt-10*** |  |
|  | **Cbg-*pccb-1*** | ***wrt-2 (CBG14131)*** | **Cbg-*sec-3*** |  |
|  | **Cbg21670** | ***wrt-3 (CBG21671)*** | **Cbg25106** |  |
|  | **Cbg-*srxa-9*** | ***wrt-4 (CBG07767)*** | **Cbg-*bus-17*** |  |
|  | **Cbr-*pcn-1*** | ***wrt-5 (CBG13429)*** | **Cbg00237** |  |
|  | **Cbr-*sax-3*** | ***wrt-6 (CBG14222)*** | **Cbg14223** |  |
|  | **Cbg27396** | ***wrt-9 (CBG16423)*** | **Cbg16422** |  |
|  | ***wrt-1*** | ***wrt-10 (CBG12987)*** | **Cbg25541** |  |
| ***P. pacificus*** | **PPA18368** | ***wrt-1 (PPA38836)*** | ***wrt-10*** |  |
|  | **Pp-*pccb-1*** | ***wrt-2 (PPA23762)*** | **PPA23764** |  |
|  | **PPA17986** | ***wrt-4iii (PDM72491.1)*** | **PPA17802** |  |
|  | **PPA06413** | ***wrt-3-5 (PPA06412)*** | **PPA0611** | ***Degenerate (multiple cysteines missing)*** |
|  | **PPA31587** | ***wrt-6 (PPA17223)*** | **PPA17221** |  |
|  | **PPA37048** | ***wrt-9 (PPA00248)*** | **PPA00251** | ***Degenerate (multiple cysteines missing)*** |
|  | ***wrt-1*** | ***wrt-10 (PPA45000)*** | **PPA18371** |  |
| ***S. ratti*** | **SRAE_X000259200** | ***wrt-1* SRAE_X000259300** | **SRAE_X000259400** |  |
|  | **SRAE_1000112500** | ***wrt-2 (*SRAE_1000112600*)*** | **SRAE_1000112800** |  |
|  | **SRAE_1000168901** | ***wrt-4 (*SRAE_1000167801*)*** | **SRAE_1000169201** |  |
|  | **SRAE_X000039300** | ***wrt-3-5 (*SRAE_X000039400*)*** | **SRAE_X000039500** |  |
|  | **SRAE_X000100400** | ***wrt-6 (*SRAE_X000100500)** | **SRAE_X000100600** |  |
|  | **SRAE_2000396500** | ***wrt-9 (*SRAE_2000396600)** | **SRAE_2000396700** |  |
|  | **SRAE_X000005500** | ***wrt-10 (*SRAE_X000005600*)*** | **SRAE_X000005700** |  |
| ***A. suum*** | **ASU_02743** | ***wrt-2 (ASU_02744)*** | **ASU_02745** |  |
|  | **ASU_01525** | ***wrt-3-5 (ASU_01526)*** | **ASU_01527** |  |
|  | **ASU_03946** | ***wrt-9 (AgB01_g641_t01)*** | **ASU_03948** |  |
|  | **ASU_09186** | ***wrt-10 (ASU_09186)*** | **ASU_09188** |  |
| ***T. canis*** | **-** | ***wrt-2 (Tcan_18550)*** | **-** | **gene desert** |
|  | **Tcan_04003.1** | ***wrt-3-5 (Tcan_04018)*** | **Tcan_04021.1** |  |
|  | **-** | ***wrt-3-5 (Tcan_07009)*** | **-** | **gene desert** |
|  | **Tcan_18250.1** | ***wrt-9 (Tcan_18248.1)*** | **Tcan_18243.1** |  |
|  | **Tcan_14303.1** | ***wrt-10 (Tcan_14314.1)*** | **Tcan_14311.1** |  |
| ***B. malayi*** | **Bm8326** | ***wrt-2 (Bm4264)*** | **Bm18002** |  |
|  | **-** | ***wrt-3-5 (Bm3920)*** | **-** | **gene desert** |
|  | **Bm11541** | ***wrt-6 (Bm7883)*** | **Bm18141** |  |
|  | **-** | ***wrt-10 (Bm7761)*** | **-** | **gene desert** |
| ***T. spiralis*** | **T01_4848.1** | ***wrt-X***  ***(T01_6149.5)*** | **T01_11095.1** |  |

**Additional File 6: *wrt-7* is nonfunctional in *C. elegans***


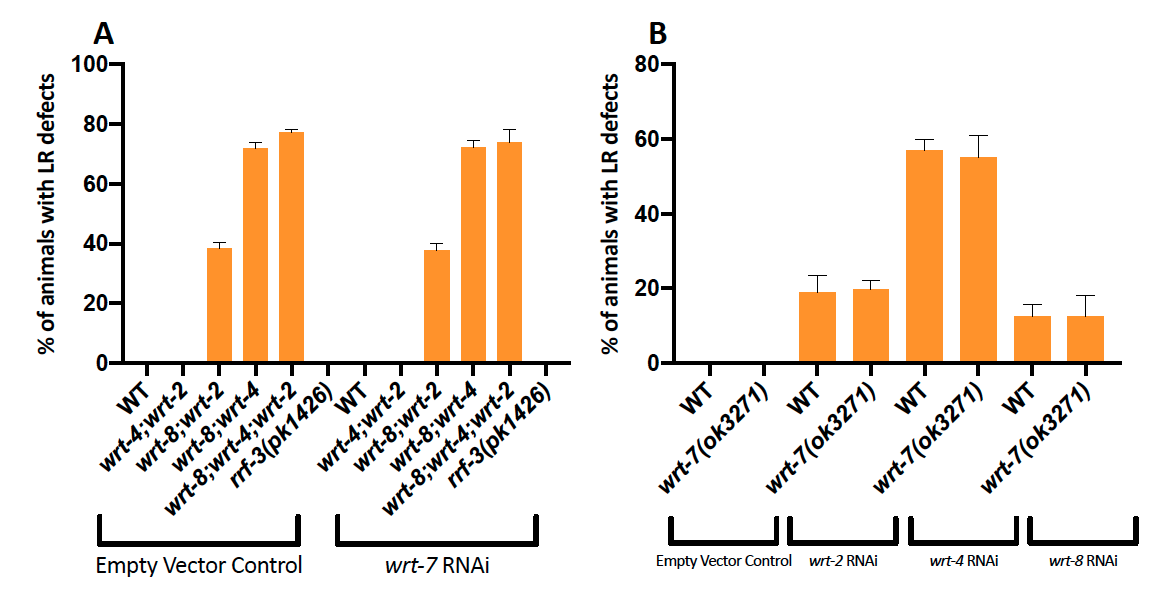


**Additional File 7: Naturally occurring variations in *wrt-7* among wild isolates of *C. elegans***


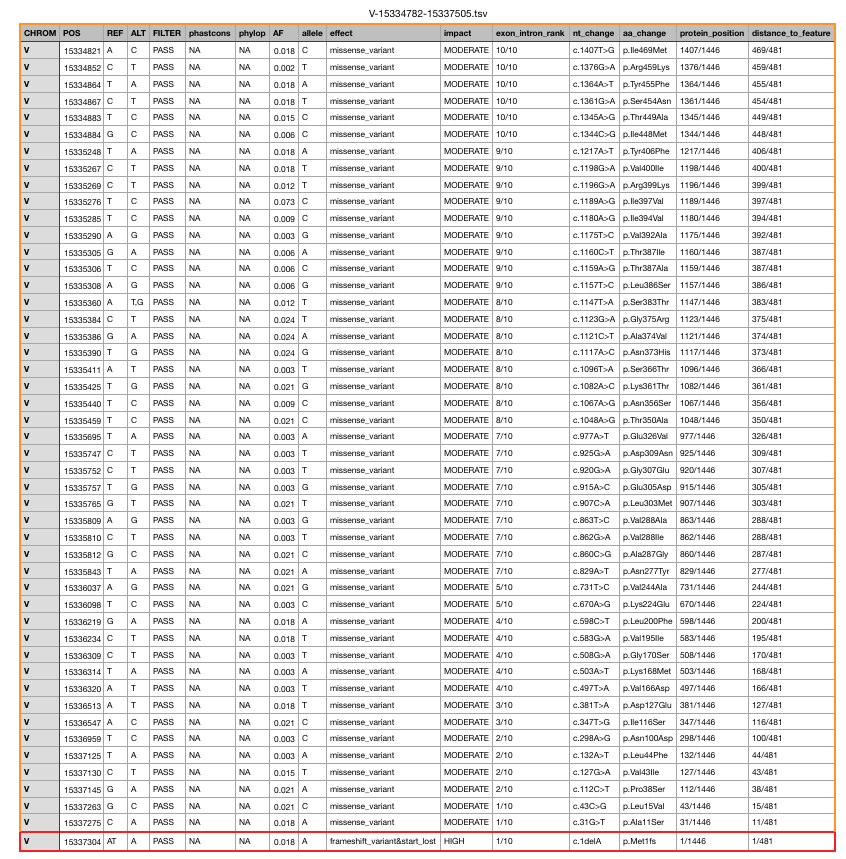


**Additional File 8: Severe morphological defects of *wrt-3* defective animals**


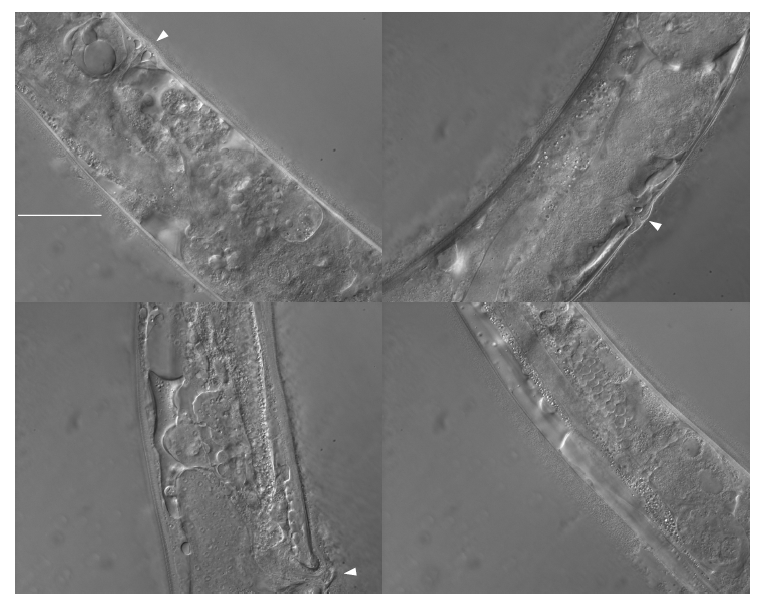


**Additional File 9: Interclade RNAi reveals no further redundancy in the Warthog family**

**Additional File 10: Deconstruction of Figure 6**

**e)**

**b)**

**h)**

**d)**

**g)**

**c)**

**f)**

**a)**

**l)**

**k)**

**j)**

**i)**

**Additional File 11: Strains and deletion alleles used in this investigation**

| **Genotype** | **Strain name** | **Mutation** | **Size of deletion** | **Wart domain affected?** |
| --- | --- | --- | --- | --- |
| **Wildtype** | N2 | n/a | n/a | n/a |
| ***him-5(e1490)*V** | CB4088 | substitution | n/a | n/a |
| ***ajm-1::gfp*** | P57657 | n/a | n/a | n/a |
| ***rrf-3(pk1426)*** | NL2099 | deletion | n/a | n/a |
| ***wrt-1(tm1417)*II** | AW1796 | deletion | 616 bp | Yes - truncated |
| ***wrt-2(ok2810)*X** | RB2125 | deletion | 500 bp | Yes - truncated |
| ***wrt-3(ok2608)*IV** | VC2083 | deletion | 688 bp | Yes - truncated |
| ***wrt-4(tm1911)*X** | AW1258 | deletion | 912 bp deletion + 5 bp insertion | Yes - truncated |
| ***wrt-5(ok670)*IV** | RB843 | deletion | 371 bp | Yes - truncated |
| ***wrt-7(ok3271)*V** | VC2584 | deletion | 701 bp | Yes - truncated |
| ***wrt-8(tm1585)*V** | AW1299 | deletion | 1256 bp | Yes - truncated |
| ***wrt-9(ok2732)*X** | RB2069 | deletion | 979 bp | Yes - truncated |

**Additional File 12: Oligonucleotides used in this investigation**

wrt-1_fwd CGTGAATTCTCATCTTCCAGCTGC 24-mer

wrt-1_rev GTCATAACCATATCTCCAACACTCAAATC 29-mer

wrt-2_fwd CATTCGCTGACCAACTGGAGGTATG 25-mer

wrt-2_rev CGACCTGCAGGCATGCAAGCTGAGGACTCCGAAAAATTTGGCAAC 45-mer

wrt-3_fwd AATCGAGAAGAAGACTTTGGGA 24-mer

wrt-3_rev GGCACAAGTTTTCTTCAAACATCAC 25-mer

wrt-4_fwd CTACGCTCCATTGCGTGCATC 21-mer

wrt-4_rev CATTCTGAACACCTTGTGGGAC 22-mer

wrt-5_fwd GGCTCAGCTCAAAAAGTGATGAT 23-mer

wrt-5_rev CTCGCCAGTGCGGATAGTAA 20-mer

wrt-7_fwd TCCTCTTCGAGAATCCACTGATC 23-mer

wrt-7_rev GTCGGCACTCTGTGGTGTAT 20-mer

wrt-8_fwd AAGAGTCATGGGCGTTTCGT 20-mer

wrt-8_rev CACAGGGTGGAAAGGTGGAC 20-mer

wrt-9_fwd CACCTCTATCATAGCTCAGAATTAAATTCC 30-mer

wrt-9_rev CATTAAGTTGCAGCGGCTGAC 21-mer
